# Interventions Designed to Improve Adherence to Growth Hormone Treatment for Pediatric Patients and Their Families: A Narrative Review

**DOI:** 10.3390/pharmaceutics14112373

**Published:** 2022-11-04

**Authors:** Selina Graham, Sophia Quirke-McFarlane, Vivian Auyeung, John Weinman

**Affiliations:** School of Cancer and Pharmaceutical Sciences, King’s College London, London SE1 9NQ, UK

**Keywords:** children, pediatric, injection device, patient choice, short stature

## Abstract

Even though growth hormone (GH) treatment is still the only active treatment option to correct growth failure and increase stature for patients with GH deficiencies, evidence has shown that non-adherence remains high. The aim of this review was to identify and review the existing interventional strategies that have been designed to address and improve adherence to GH treatment for pediatric patients and their families. An extensive search of several electronic databases was undertaken to identify relevant interventional studies, published in English, between 1985 and 2021. Additional search strategies included hand-searching topic review articles to identify eligible studies. Articles were screened against the inclusion eligibility criteria and data on sample characteristics, intervention features, and key findings was extracted. A total of fifteen interventional studies were included in the review. The interventions identified were divided into two broad categories: novel injection devices, and patient choice of device. In conclusions, this review acknowledges that there is a lack of evidence-based, theory-driven intervention strategies, designed with the purpose of optimizing treatment adherence and improve clinical and psychosocial outcomes.

## 1. Introduction

Since the availability of recombinant human growth hormone (rhGH) treatment in the 1980s, patients with growth hormone deficiencies have been prescribed a daily supplementary bio-synthetic injection of rhGH in order to replace the deficient hormone [1,2,3,4]. RhGH treatment has been approved in Europe and the USA for several different conditions associated with short stature, including Growth Hormone Deficiency (GHD), Small for Gestational Age (SGA), Turner Syndrome (TS), Prader-Willi Syndrome (PWS), Russell Silver Syndrome (RSS), Short stature HomeobOX-containing gene (SHOX) deficiency, Idiopathic short stature (ISS), Chronic Renal Insufficiency (CRI), and Noonan Syndrome (NS) [1,3,5,6,7,8]. The appropriate daily use of the prescribed rhGH treatment by the pediatric patient and their family is imperative throughout the treatment pathway for optimal clinical benefit to be realised [5,9].

However, even though rhGH treatment is still the only active treatment option to correct growth failure and increase stature for patients with growth hormone deficiencies, evidence has shown that non-adherence remains high [2,8,10,11]. Treatment adherence has been specifically defined as ‘the extent to which an individual’s behaviour is in accordance with the agreed recommendations from their health professional’, with respect to three distinct phases: initiation (i.e., starting treatment), implementation (i.e., incorporating treatment into pattern of life, in accordance with prescribed dosing regimen), and discontinuation (end of treatment) [12,13,14,15]. A systematic review that examined the prevalence of non-adherence amongst the various clinical indications for rhGH treatment found that up to 82% were non-adherent to their treatment as prescribed [2]. Suboptimal adherence affects the long-term clinical effectiveness of the treatment for the patient and impacts considerably upon the healthcare provider and healthcare system, in terms of resources and healthcare costs [2,3,13,16,17,18,19]. Given this substantial impact, treatment non-adherence has become an increasingly important health issue amongst research and clinical practice [17].

Adherence to rhGH treatment, however, is a complex in nature and is driven by a myriad of patient-related, healthcare professional-related and healthcare system-related factors [19,20,21], as acknowledged by a recent systematic review [10]. Amongst the included studies of the review, the range of barriers to adherence were found to center around: the discomfort and pain associated with administering the daily injection; the skill and self-efficacy of self/parental administration; concerns about the treatment, i.e., long-term effects; and the quality of the HCP–parent/caregiver relationship [10,22,23,24,25,26,27]. Numerous interventions have been designed and developed in an effort to alleviate these barriers to adherence and optimize the use of prescribed rhGH treatment amongst pediatric patients and their families [2]. The aim of this review is to provide a comprehensive overview of the nature of these existing interventional strategies.

## 2. Methodology

### 2.1. Search Strategy

An extensive search was undertaken of electronic databases, which included the Cochrane Library, Excerpta Medica dataBASE (EMBASE), PsycINFO, Medline, International Pharmaceutical Abstracts, Web of Science, Cumulative Index to Nursing and Allied Health Literature (CINAHL), and Applied Social Sciences Index and Abstracts (ASSIA), using a comprehensive set of search terms (see Supplementary Table S1) to identify relevant published interventions. Additional search strategies included hand-searching topic review articles to identify eligible studies. The last online search was conducted on 31 December 2021. Manual searches via journals, books chapters, and reference lists of relevant articles were also undertaken to identify any additional records. The search was limited to full-text studies published in the English language, between 1985 and 2021. The decision to use this search period was based on the licensing of recombinant human growth hormone (somatropin) for the treatment of GHD in 1985 [3].

### 2.2. Search Terms

The key search terms (alternatives and synonyms) were tailored to comprise four main conceptual emphases, identified from the research question: (i) population-related terms, i.e., “child*”, “paediatric”/”pediatric”, (ii) treatment-related terms, i.e., “growth hormone”, “somatropin”, “injection”, (iii) adherence-related terms, i.e., “adherence”, “persistence”, “compliance” and (iv) study design-related terms, i.e., “intervention”, “control*” (see Supplementary Table S1).

### 2.3. Inclusion/Exclusion Criteria

Interventional studies were included if they met the following criteria:

(i) Patients aged ≤18 years, prescribed with rhGH treatment.
(ii) Pediatric patients with a diagnosis of the various forms of short stature or growth failure (observant of pediatric patients with a diagnosis of GHD) or parent/caregivers.
(iii) Primary or secondary aim to assess/monitor and improve the level of adherence to rhGH treatment.
(iv) Randomized controlled trials (RCT) and non-RCT (prospective cohort and retrospective cohort studies), cross-sectional studies or longitudinal studies.
(v) Interventions with a parallel group design where treatment group is compared with a clearly defined control/comparator group, or within-subject pre-post test.
(vi) Hospital or home-based, patient/parent-facing or HCP-facing; self-led, parental-led or HCP-led interventions.
(vii) Standardized measure of treatment adherence (both validated/non-validated methods) explicitly identifiable.
(viii) Results from a standardized measure of adherence explicitly extractable as a primary outcome of intervention or as a secondary outcome.
(ix) Full text studies in English published between 1985 and 2021.

### 2.4. Data Collection and Extraction

First, all articles were manually screened by two of the authors, based on their title and abstract, to determine eligibility according to the inclusion criteria (S.McF. and S.G.). Relevant full-text studies were assembled and evaluated for their eligibility. Four study authors were contacted directly for further information, to clarify aspects of their methodology and/or to retrieve access to full-text papers in which to determine eligibility. Co-authors (V.A. and J.W.) undertook partial screening to validate the study selection and data extraction process; any reviewer uncertainties or disagreements were discussed until consensus was met. Studies that did not fulfil the criteria were omitted throughout the process, with accompanying reasons for exclusion (see Supplementary Table S2). Data from each article were extracted using a standardized data extraction form (Cochrane Consumers and Communication Review Group Data Extraction Template), relating to the: (1) Study details, (2) Participant characteristics, (3) Intervention Features, (4) Adherence Measurement details, and (4) Key findings (see Supplementary Table S3).

## 3. Results

Seventy-nine full-text articles were assessed for eligibility, of which fifteen were identified as meeting the inclusion criteria and included in the review.

### 3.1. Study Characteristics

Table 1 summarises the study characteristics of the 15 interventional studies included in this review [24,28,29,30,31,32,33,34,35,36,37,38,39,40,41]. The interventional studies were conducted in France [39], Italy, the UK [36,38,41], Spain [28,37], Germany [24], The Netherlands [40], the USA [32], Japan [33], and Mexico [29], whilst one study [30] was conducted internationally. Included in this review were six prospective observational studies [24,31,34,37,40], five retrospective observational studies [28,32,35,36,38], one longitudinal observational study [29], one observational survey study [30], one prospective open-label study [39], one retrospective longitudinal survey study [33], and one prospective cross-sectional survey study [41]. The total sample size across all 15 included studies was 6673 patients (mean = 444.9; range = 30–4093). All interventions designed to improve adherence to rhGH treatment were found to be inclusive of the various endocrine conditions treated with rhGH treatment (see Table 1). One study [38] did not explicitly specify the patients’ condition(s). In the studies that reported rhGH indications (*n* = 14), the GHD population accounted for the majority in nine studies [24,29,31,32,35,36,37,41,42] of which, in three of the studies, GHD accounted for 100% of the sample group [33,34,40]. In two studies [28,39], the SGA population made up the majority, followed closely by pediatric patients with GHD. The mean age of participants across 12 studies [24,28,29,31,32,33,35,36,37,38,39,40] was 9.37 years (SD = 2.00); three of the 15 studies [30,34,41] reported the median age of participants (11, 10, and 9.3 years old, respectively). Amongst the included studies, 58.4% of participants were male (range = 50.0–75.8%).

### 3.2. Interventions Developed to Improve Adherence to rhGH Treatment

The interventions identified to specifically address and improve non-adherence to rhGH treatment can be divided into two broad categories: ‘novel injection devices’ and ‘patient choice’ of device.

Whereas ‘novel injection devices’ involves the development of new rhGH injection delivery devices, for example, the advancement of new shapes and sizes of device, methods of injection (e.g., manual, needle-free, automatic, electronic), and new methods for preparation and reconstitution (e.g., pre-filled, needle shields) (see Table 1), ‘patient choice’, as labeled by the researchers, is defined as the opportunity provided by the healthcare professionals, for both patients and their caregivers to make an informed and shared choice of injection device, upon the initiation of rhGH treatment [5,33,41]. Although the two identified categories of interventions have defined differences, it is important to note that they are not mutually exclusive.

### 3.3. Novel Injection Devices

Thirteen studies were found to explore the impact of novel injection devices on adherence, of which nine studies [24,28,29,31,34,35,37,40,42] investigated a hidden-needle electronic auto-injector—the easypod™ device and e-Health feedback system (Merck Serono International S.A., Geneva, Switzerland). Four of these studies [29,31,37,40] were part of the Easypod Connect Observational Study, which was a prospective international 5-year investigation monitoring adherence to rhGH treatment in patients receiving rhGH via the easypod™ system. Three studies [32,36,38] examined a non-invasive needle-free injector; the Cool.click™ device (Merck Serono International S.A., Geneva, Switzerland) in one study [32], and the ZomaJet^®^ device (Ferring Pharmaceuticals, London, UK) in two studies [36,38]. One study [39] assessed a prefilled, multidose, disposable injector pen—the NordiFlex^®^ device (Novo Nordisk A/S, Bagsvaerd, Denmark).

### 3.4. Patient Choice

The impact of offering patients and their families a choice of injection device upon initiation of rhGH treatment on treatment adherence was explored in two observational studies [33,41].

### 3.5. Measurement of Adherence

Of the 15 interventional studies included within the review, the assessment/monitoring of adherence was identified as a primary outcome in 12 studies and a secondary outcome in three (see Supplementary Table S3). All interventional studies included within the review explored adherence within the implementation phase; one study also explored persistence to treatment [38].

Various methods across the interventional studies were used to measure adherence to rhGH treatment (see Table 1). Five studies [28,29,31,34,37] used an electronic monitoring device, one study [41] used ampoule counts, one study [39] used patient/parent diaries, one study used physician reports [32], and one study [33] used self-report questionnaires. Two studies [36,38] used issued, renewed, or redeemed rhGH prescriptions. Four studies used a combination of measures as a means of measuring treatment adherence: two studies [30,35] used an electronic monitoring device in conjunction with a patient/parent self-report survey, one study [24] used an electronic monitoring device in conjunction with a clinical kit software and one study [40] used an electronic monitoring device combined with physician data entry of outcome measures. The observation time periods to assess and monitor adherence similarly varied across the studies, ranging from 6 weeks [39] to 4 years [37] (see Table 1). In one study, the follow-up period [35] was variable based on each patient according to clinical practice.

Similarly, the level of adherence was assessed across the studies in several ways. For the majority of studies [24,28,29,31,34,37,40,42], adherence was calculated as the percentage/proportion of injections received with respect to planned injections. In one study [41], adherence was calculated by comparing the number of ampoules of GH used against expected ampoule usage. In two studies [36,38], adherence was determined using the proportion of days covered (PDC) measure (the ratio of the number of days a patient had access to viable rhGH device-heads (quantity of device-heads delivered × length of time each head should last) to the number of days they were prescribed rhGH treatment during the treatment period) as it was considered to provide a more conservative estimate of adherence compared to the medication possession ratio (MPR). Two studies [32,33] did not report the adherence calculation utilized. For the two studies [30,35] that collected adherence via an electronic monitoring device and a patient/parent self-report survey, recorded adherence (via the electronic monitoring device) was calculated as the imputation of missing period(s) using non-missing period(s) [30] and the percentage of injections received (days) with respect to planned injections. In one of the studies [30], reported adherence (via the survey) was calculated by the number of missed injections [e.g., 0; 1–3; 4–6; 7–9; ≥10], however the other study [35] did not specify how reported adherence was calculated.

Adherence was also quantified by a range of cut-offs thresholds across the studies. In two studies [37,39], the adherent population was defined as those with ≥85% adherence to prescribed treatment. In two studies [30,34], adherence was defined as those with ≥92% adherence to prescribed treatment. In two studies [36,38], a PDC score of >0.8 indicated that a patient was highly adherent. One study [24] used the following cut-offs proposed by Cutfield et al. [23]: good/high adherence, missed ≤1 dose per week (85.7–100% proportion injected); medium adherence, missed > 1 but < 3 doses per week (57.1–85.7% proportion injected); and poor/low adherence, missed ≥3 doses per week (<57.1% proportion injected). One study [28] used slightly different categories to classify adherence: excellent adherence (>95%); good adherence (85–95%); fair adherence (75–85%); and poor adherence (<75%). In one study [40], adherence level was different for each time-point: year 1, ≥98%; year 2, ≥91%; first two years, ≥78%. In one study [39], the “absolutely adherent” population was defined as those who missed no daily rhGH dose during the 6-week study period. In one study [32], the adherent population was defined as those who missed <3 doses per month. Adherence cut-off thresholds were not defined in five studies [29,31,33,35,41].

### 3.6. Adherence Rates

The studies observed marked variations in the reported levels of adherence that ranged from 56.7% [34] to 94.5% [37] (see Table 1). The adherence rate for pediatric patients receiving rhGH via the electronic auto-injector device was >80% in the majority of studies [24,28,29,31,34,35,37,40,42], with rates varying between 56.7% [34] to 94.5% [37]. For patients receiving rhGH via needle-free devices [32,36,38], studies reported levels of adherence between 58% [38] and 84.6% [32]. The single study assessing adherence via the injection pen device [39,40] reported that 70.6% of patients were classified as “absolutely adherent”. The studies [33,41] examining patient choice of device, however, had conflicting adherence findings; whilst one study [33] reported adherence levels for the patient choice group and non-patient choice group as 92.9% and 66.7%, respectively; the other study [41] found that offering patients a choice of device had no significant effect on adherence, although overall adherence remained high at >85%.

## 4. Discussion

The primary aim of this review was to identify and evaluate the existing interventional strategies that have been designed to address and improve adherence to rhGH treatment for pediatric patients and their families. It is important to note that the review forms part of a wider PhD thesis [43]. From an extensive search, 15 relevant interventional studies were identified with a primary or secondary aim to assess/monitor and improve the level of adherence to rhGH treatment.

The majority of interventions designed to improve adherence to rhGH treatment centered around the development of new delivery devices (*n* = 13), namely, an electronic auto-injector and digital feedback system [24,28,29,31,34,35,37,40,42], needle-free injectors [32,36,38], and an injector pen [39]. The primary focus of these interventions was to enhance the experience of daily injections for patients and their families, by simplifying and improving the drug delivery process, therefore alleviating injection discomfort, pain and anxiety, reducing treatment burden, and increasing treatment tolerability. Furthermore, the e-Health feedback system associated with the electronic auto-injector enabled ongoing monitoring and timely input, if necessary. Two interventions [33,41] targeted the choice of injection device as a means to accommodate individual needs and preferences at initiation and maximize treatment ownership and acceptability; as national clinical guidelines have emphasized: ‘The choice of product should be made on an individual basis after informed discussion between the responsible clinician and the patient and/or their carer about the advantages and disadvantages of the products available, taking into consideration therapeutic need and the likelihood of adherence to treatment’ [3]. Notably, within the selection process of relevant interventions, a large number of studies were initially identified, which included usability studies of novel injection devices [44,45,46,47,48,49,50,51,52,53,54,55,56,57,58,59,60] as well as studies that explored reduced injection frequency via long-acting sustained-release rhGH formulations [52,53,54,61,62,63]. Although many of these studies made reference to adherence (e.g., stating that the intervention “may improve” [50,51], “should help to increase” [44,45], “is likely to improve” [26], or “may facilitate” [46] adherence to rhGH treatment via increased acceptance, tolerability, and therapeutic flexibility), these individual interventional studies did neither address nor assess treatment adherence within the body of the content, and therefore were not included within the review (see Supplementary Table S2). Regarding these interventions, in particular, long-acting rhGH preparations, it is recommended that future research further explore the impact of reducing the frequency of injections on treatment adherence and clinical health outcomes for pediatric patients and their families.

Of the included interventional studies that aimed specifically to address and improve adherence to rhGH treatment, there was, however, considerable methodological heterogeneity. A variety of methods were used to measure adherence, ranging from self-report questionnaires to electronic monitoring devices. Across the studies, the calculation used to determine adherence were also varied, including assessing the percentage/proportion of injections received with respect to planned injections. Furthermore, the cut-off thresholds for adherence differed notably across the studies, ranging from ≥85% [28,37,39] to missed <3 doses per month. Moreover, in one study [40], adherence level was different for each time-point: year 1, ≥98%; year 2, ≥91%; and first two years, ≥78%. The variability across studies may indeed explain some of the differences in the reported rates of treatment adherence, which ranged from 56.7% [34] to 94.5% [37]. Due to the heterogeneous nature of the included studies, the collation and evaluation of methodologies, results, and conclusions across interventional studies was complicated [17]. It is suggested that researchers continue to work towards a strategy of standardization within adherence research, to facilitate more clear-cut interpretations and comparisons of future interventional studies.

More specifically, ‘patient choice’, as labeled by the authors of the studies, is defined as the opportunity provided by the HCPs (within the initiation consultation) for patients and their caregivers to make an informed choice of rhGH injection device [5,33,41]. It is important to note, however, that the process of injection choice at the initiation of rhGH treatment is often made collaboratively by the pediatric patient and their parent/caregiver. The clarity and differentiation of who is making the device choice, in addition to who is reporting adherence, was not always present within several relevant studies [30,33,35,39] yet is crucial to our understanding of treatment adherence, thereby presenting a limitation. It is therefore strongly recommended that the injection choice process and subsequent reporting of adherence is clarified within future studies, to ensure transparency and to facilitate a more unequivocal comparison of studies.

In addition, it was beyond the scope of our review to examine a number of important variables, such as age/psychosocial maturity of the patient, treatment duration, diagnosis, or severity of diagnosis. On looking specifically at the age groups and underlying diagnoses within the selected studies, no obvious patterns of difference were detected. To explore these areas further is important, and worthy of future research.

Further, the reporting of effect sizes was lacking across the included interventional studies. This prevented an accurate measure of the effectiveness of the interventions. The routine use of effect sizes has been found to be generally limited to meta-analyses for combining and comparing estimates from different studies, and is increasingly uncommon amongst original reports of research [64]. It is recommended that future primary studies calculate and report effect sizes, in addition to significance testing in order to quantify the magnitude of the effect of the intervention and facilitate the decision whether a clinically relevant effect has been found.

Amongst the interventional studies, improved adherence amongst the endocrine population groups were generally reported [37]. Although these levels of treatment adherence appear promising, it is important to note that these studies were largely observational in nature, and therefore require a more rigorous evaluation, e.g., via a randomized controlled trial (RCT), to reliably determine the effectiveness of the new interventions [65]. To date, there do not appear to be any RCTs currently in this area.

According to the Medical Research Council’s (MRC) framework for developing and evaluating complex interventions [66], it is fundamental that interventions are based on a body of empirical research. Amongst the included studies, there was, however, little available evidence that the interventions were directly informed by prior analysis of the determinants of non-adherence. Furthermore, it was expected to find a higher proportion of interventional studies that used an appropriate theory-based approach within their design to improve treatment adherence. The guidance provided by the MRC framework emphasizes the importance of identifying and developing a theoretical understanding of the likely mechanisms of change, when developing complex interventions [66]. Yet, across all the interventional studies, theoretical frameworks were not explicitly referenced. The lack of transparency in study reporting with regard to the evidence and theoretical base is an issue that limits future replication, as well as challenges the identification of the techniques that have been effective in changing behavior. The lack of evidence-based and theory-driven intervention strategies highlights an important avenue for future research efforts. Moreover, the increasing use of checklists such as the Template for Intervention Description and Replication (TIDieR) presents an opportunity to improve the quality of intervention reporting across adherence research [43].

Lastly, previous research has made evident the wide range of potentially modifiable factors that influence low levels of treatment adherence amongst children with growth hormone deficiencies and their families [2,10]. Although the interventions identified within this review could potentially target a number of appropriate drivers of non-adherence, namely the discomfort and pain associated with daily injections [27] or the lack of choice of injection device within clinical practice [25], it is clear that the identified interventions are not capable of targeting the breadth of factors found to be associated with treatment non-adherence. Targeting individual determinants is not considered an effectual or cost-effective way in which to address non-adherence [67,68,69] and bring about behavior change. It is proposed, therefore, that future research acknowledges that one-size does not fit all and that changing complex behaviors, such as adherence, requires a much broader approach. To increase adherence to rhGH treatment, there is a need for effective, multi-faceted intervention strategies designed with the ability to target and address the wide range of different factors that have been found to influence adherence amongst this population [2,10,17]. As Haynes et al. (2008) observed, ‘the majority of interventions that were found to be effective for long-term care were complex and targeted multiple adherence determinants’ [70].

## 5. Conclusions

This narrative review presents a comprehensive overview of the different existing interventional strategies that have been developed to optimize adherence to rhGH treatment amongst pediatric patients and their families, which has unique value within the current GHD literature. It is recommended that future research starts to focus on designing, developing, and implementing new, evidence-based and theory-driven intervention strategies, with the purpose to optimize treatment adherence and improve clinical and psychosocial outcomes.

## Figures and Tables

**Table 1:** Summary Data Extraction Table.

Study Details			Participant Characteristics		Intervention	Adherence Measurement		Key Findings
Author and Publication Year	Study Design	Sample Size	Age	Clinical Indication of GH Therapy. N (%)		Adherence Measure	Observation Time Period	
Electronic auto-injector								
Arrabal Vela et al. (2018) [28]	Retrospective, longitudinal descriptive study	30 pediatric patients	Total mean age = 6.09 (4.92–7.25) years	SGA = 17 (56.6%); GHD = 11 (36.6%); TS = 2 (6.7%)	easypod^TM^ device	Electronic monitoring	12-months	Mean treatment adherence was 92.3%. According to the adherence categories: 60% of the patients were defined as excellent compliers, 30% good compliers, 3.3% fair, and 6.7% poor compliers
Blanco-López et al. (2020) [29]	National, multicenter, longitudinal observational study	147 pediatric patients	Total mean age = 9.96 ± 3.41 years	GHD = 118 (80.3%); SGA = 24 (16.3%); TS = 5 (3.4%)	easypod^TM^ device	Electronic monitoring	3 months, 6 months, 1 year, 2 years, 3 years	Mean adherence was: >80%. 90.4% (*n* = 146) at the 3 month follow-up, 87.4% (*n* = 143) at the 6 month follow-up, 85.7% (*n* = 135) at the 1-year follow-up, 83.9% (*n* = 97) at the 2-year follow-up, 84.5% (*n* = 39) at the 3-year follow-up
Bozzola et al. (2011) [30]	Multicenter, multinational, observational survey study	824 pediatric patients	Total median age (range) = 11 (1–18) years	GHD = 543 (65.9%); TS = 80 (9.7%); SGA = 125 (15.2%); CRF = 14 (1.7%); Other = 56 (6.8%)	easypod^TM^ device	Electronic monitoring (recorded adherence) in conjunction with a patient/parent self-report survey (reported adherence)	3-months	Recorded adherence: According to the recorded adherence data, 87.5% of children were adherent to treatment over the 3-month period. Month 1 = 90.5%; Month 2 = 87.1%; Month 3 = 88.9% 51.4% (397/772) of children were recorded to have missed one or more injections over the 3-month period. Reported adherence: According to self-reported data, 90.2% (*n* = 607/673) of children were adherent over 3 months; 51.5% (*n* = 421/817) missed ≥1 injection over this period
Centonze et al. (2019) [31]	Prospective, longitudinal, observational study	73 treatment-naïve pediatric patients	Total mean age = 9.78 ± 3.20 years	Idiopathic GHD = 70 (95.9%); Organic GHD = 2 (2.7%); Congenital GHD = 1 (1.4%)	easypod^TM^ device	Electronic monitoring	1 years, 2 years, 3 years	Mean adherence was >85% over the 3-year follow-up period: 1-year follow up = 88.5% (*n* = 65), 2-year follow-up = 86.6% (*n* = 40) 3-year follow-up = 86.5% (*n* = 18)
Hartmann et al. (2013) [24]	Prospective observational study	75 pediatric patients	Total mean age = 12.5 ± 3.5 years	GHD = 48 (64.0%); SGA = 18 (24.0%); TS = 6 (8.0%); CRF = 3 (4.0%)	easypod^TM^ device	Electronic monitoring in conjunction with a clinical kit software	The average observation time was 343 ± 201 days (range 28–1034 days)	The mean (±SD) rhGH treatment adherence rate of all patients was 91.2 ± 12.2%. According to the definitions of Cutfield et al. [23] 2.7% of all patients had poor compliance, 18.7% had medium compliance, and 78.7% had good compliance. 77.1% of patients with GHD showed good compliance. Approximately 90.0% of SGA patients were categorized as good compliers (10.0% medium, 10.0% poor). Approximately 50.0% of TS patients showed good compliance, while the remaining 50% were categorized as medium compliers. Approximately 100% of CRF patients showed good compliance
Loche et al. (2016) [34]	Prospective observational study	79 pediatric patients	Median age at enrolment (interquartile range) = 10 (9–12) years	GHD = 100%	easypod^TM^ device	Electronic monitoring	1 year	56.7% of the patients were considered to be fully (≥92%) adherent to their treatment throughout the 1-year study period.
Maggio et al. (2018) [35]	Retrospective, observational monocentric study	40 pediatric patients	Total mean age = 11.2 ± 2.3 years	Isolated GHD = 26 (65%); SGA = 9 (22.5%); TS = 5 (12.5%)	easypod^TM^ device	Electronic monitoring (recorded adherence) in conjunction with a patient/parent self-report survey (reported adherence)	Data were collected at baseline, (before the treatment start), and after appropriate follow-up, which was variable for each patient, according to clinical practice	Recorded adherence: The mean treatment adherence was 92.20%. 1-year (96.0%, *n* = 13) 2–4 years (94.7%, *n* = 17) 4 years (83.9%, *n* = 10). [Questionnaire Evaluation] Reported adherence: Comparing the electronic evaluation of adherence, with the questionnaire answers, 26 patients (65.0%) referred a lower number of skip doses compared to what registered by easypod™, on the contrary 5 patients (12.5%) referred a higher number. Thus, 9 patients (22.5%) referred a skip doses number equal to what registered by the electronic device. In general, the mean skip doses number referred to parents was 1.3 doses monthly, although increasing until 2.5 doses monthly considering easypod™ data
Rodríguez Arnao et al. (2019) [37]	National, multicenter, prospective observational study	238 pediatric patients	Total mean age at inclusion (±SD) = 9.0 ± 3.3 years; Total mean age at treatment initiation = 7.9 ± 3.2 years.	GHD = 144 (60.5%); SGA = 86 (36.1%); TS = 8 (3.4%)	easypod^TM^ device	Electronic monitoring	6 months, 1 year, 2 years, 3 years and 4 years	Mean overall adherence was 94.5%. Adherence was higher than 90% in all follow-up visits: 97.5% after 6 months (*n* = 234) 95.3% after 1-year (*n* = 232) 93.7% after 2 years (*n* = 174) 94.4% after 3 years (*n* = 84) and 95.5% after 4 years of treatment (*n* = 25)
van Dommelen et al. (2018) [40]	Prospective observational study	95 treatment naïve pediatric patients	Mean age = 6.3 ± 2.1 years	Idiopathic isolated GHD = 100%	easypod^TM^ device	Electronic monitoring combined with physician data entry of outcome measures	2 years	In the first year: 32 children (34%) had high adherence and 63 children (66%) had low adherence. In the second year: 50 children (53%) had high adherence and 45 children (47%) had low adherence. For the first two years: 68 children (72%) had high adherence and 27 children (28%) had low adherence.
Needle-free injector								
Desrosiers et al. (2005) [32]	Retrospective cohort study	631 pediatric patients	NFDS patients: Total mean age: 10.6 ± 3.9 Needle device patients: Total mean age: 10.1 ± 3.9	NFDS patients: Idiopathic GH deficiency = 218 (78.7%); TS = 16 (5.8%); Organic GH deficiency = 7 (2.5%); Other dysmorphic = 21 (7.6%); SGA = 6 (2.2%); PWS = 2 (0.7%); Neurosecretory dysfunction = 4 (1.4%); Noonan syndrome = 1 (0.4%); Chondrodystrophy = 1 (0.4%); Congenital adrenal hyperplasia = 1 (0.4%); Needle device patients: Idiopathic GH deficiency = 164 (72.9%); TS = 16 (7.1%); Organic GH deficiency = 19 (8.4%); Other dysmorphic = 3 (1.3%); SGA = 7 (3.1%); PWS = 7 (3.1%); Neurosecretory dysfunction = 3 (1.3%); Noonan syndrome = 3 (1.3%); Chronic kidney disease = 1 (0.4%); Genetic GH deficiency = 1 (0.4%); Hypophosphatemia rickets = 1 (0.4%)	Cool.click^TM^ device	Physician report	24 months	Adherence was high in both the Cool.click device (84.6%) and needle and syringe (76.3%) cohorts. Compared to patients using the Cool.click device, significantly more patients using needle and syringe missed over one-half of their prescribed GH dose (6% vs. 13.4%, respectively, *p* = 0.002)
Michaelidou et al. (2019) [36]	Retrospective longitudinal study	1 year treatment cohort: 52 pediatric patients 3 year treatment cohort: 22 pediatric patients	1 year treatment cohort: Total mean age = 8.50 ± 3.78 years 3 year treatment cohort: Total mean age = 7.21 ± 3.68 years	1 year treatment cohort: GHD = 34 (65.4%); TS = 5 (9.6%); Other = 13 (25.0%) 3 year treatment cohort: GHD = 17 (77.3%); TS = 2 (9.1%); Other = 3 (13.6%)	ZomaJet^®^ device	Issued, renewed, or redeemed rhGH prescriptions	3 years	According to the 1-year data, 30 of the 52 patients (57.7%) were classified as adherent, whilst the remaining 22 patients (42.3%) were classified as less adherent. According to the 3-year data, 14 of the 22 patients (63.6%) were classified as adherent, whilst the remaining 8 patients (36.4%) were classified as less adherent
Spoudeas et al. (2014) [38]	Retrospective observational study	4093 pediatric patients	ZomaJet^®^ device: Total mean age = 8.4 ± 4.0 years. Needle-based devices: Total mean age = 9.7 ± 4.3 years	ZomaJet^®^ device: Mixed conditions treated with rhGH Needle-based devices: Mixed conditions treated with rhGH	ZomaJet^®^ device	Issued, renewed, or redeemed rhGH prescriptions	3 years	Adherence was examined in patients using ZomaJet^®^ device = 728 (17.8%). Adherence: 424 of 728 ZomaJet^®^ using patients (58%) were classified as adherent (PDC 0.8–1.8). Additionally, 175 of the 424 adherent patients (24%) were classified as over adherent (PDC > 1.8) Persistence: Mean persistence was significantly longer in patients using ZomaJet^®^ than patients using needle-based devices (599 days vs. 535 days, respectively; *p* < 0.001).
Injector pen								
Tauber et al. (2013) [39]	Prospective, multicentre, open-label study	103 pediatric patients	Total mean age = 11.7 ± 2.9 years	SGA = 51 (49.5%); GHD = 43 (41.7%); TS = 9 (8.7%)	NordiFlex^®^ device	Used patient/parent diaries	6-weeks	After the 6 week study period, 65/92 patients (70.6%) were classified as “absolutely adherent”. Additionally, 13/92 patients (14.1%) had skipped only one GH injection during the 6 week period
Patient Choice								
Gau & Takasawa (2017) [33]	Retrospective, longitudinal survey study	46 pediatric patients	Mean age = 7.70 ± 3.12 years	Isolated and idiopathic GHD = 100%	Patient choice of an injection device	Self-report questionnaires	3 years	Over the 3-year period, the non-patient choice group missed significantly more injections compared to the all patient choice group (33.3% vs. 7.1%, respectively, *p* = 0.042)
Wickramasuriya et al. (2006) [41]	Prospective cross-sectional study	125 treatment-naïve pediatric patients	Median age (range) = 9.30 (1.0–18.3) years	GHI = 69 (55%) [of which 29 were post-oncology and 4 with organic GHI due to midline defects (septo-optic dysplasia)]; TS = 16 (13%); SGA = 10 (8%); Chronic renal insufficiency = 8 (7%); PWS = 3 (2%); Others = 19 (15%)	Patient choice of an injection device	Ampoule counts	3 years	Adherence assessed in 50 (40%) children who received GH by hospital prescription and home delivery, in whom uptake of ampoules could be determined: Median adherence for all devices was 95% (range 84–105%), with 96% (range 93–100%) for needle-free devices and 87% (range 84–105%) for needled devices. This compares to a median adherence of 88% for needle-free devices (only 1 device available) and 91% (range 86–101%) (3 devices) for needled devices for those patients (*n* = 115) who had not been offered free choice of GH device but were having hospital prescription with home delivery.

Abbreviations: CRF, chronic renal failure; GH, growth hormone; GHD, growth hormone deficiency; GHI, growth hormone insufficiency; NFDS, needle-free delivery system PDC, proportion of days covered; PWS, Prader-Willi syndrome; rhGH, recombinant human growth hormone; SD, standard deviation; SGA, small for gestational age; TS, Turner syndrome.

## Data Availability

Supporting information for this publication can be downloaded at Supplementary Materials.

## Supplementary Materials

The following supporting information can be downloaded at: https://www.mdpi.com/article/10.3390/pharmaceutics14112373/s1, Table S1: Search term strategy—keyword alternatives and synonyms; Table S2: Main reasons for study exclusion table; Table S3: Full data extraction table. (a) Study details and participant characteristics, (b) Study details and intervention features, (c) Study details and adherence measurement, (d) Study details and key findings.
