# Supplementary material for: Interventions Designed to Improve Adherence to Growth Hormone Treatment for Pediatric Patients and Their Families: A Narrative Review
Source: Pharmaceutics. 2022 Nov 4;14(11):2373. doi: 10.3390/pharmaceutics14112373 (PMC9695555; doi:10.3390/pharmaceutics14112373)
Supplement: Supplementary file 1 [file pharmaceutics-14-02373-s001.zip › pharmaceutics-1964068-supplementary.pdf]

Supplementary Material

Supplementary Table S1 Search term strategy - keyword alternatives and synonyms

**Concept group 1**<br><br>**Population-related terms**	**Concept group 2**<br><br>**Treatment-related terms**	**Concept group 3**<br><br>**Adherence-related terms**	**Concept group 4**<br><br>**Study design-related terms**
female<br>male<br>girl<br>boy<br><br>child*<br>p?diatric<br>infant<br>minor<br>juvenile<br>youth<br>dependent<br>human<br><br>prepubert*<br>pre-pubert*<br>preadolescen*<br>pre-adolescen*<br>preteen*<br>pre-teen*<br>preschool*<br>pre-school*<br>pubert*<br>adolescen*<br>teen*<br><br>patient*<br><br>parent*<br>mother<br>father<br>guardian<br>carer<br>caregiver<br>care-giver	growth<br>growth hormone<br>growth-hormone<br>GH<br>human growth hormone<br>HGH<br><br>somatotro?in<br>somatotro?ic<br>pituitary<br><br><br>treatment<br>therapy<br>replacement<br><br>medic*<br>synthetic<br>recombinant<br>rGH<br>rhGH<br><br>precri*<br><br>somatropin<br>Genotropin<br>Saizen<br>Zomacton<br>NutropinAq<br>Norditropin<br>Omnitrope<br>Humatrope<br><br>regim*<br>adminst*<br>inject*<br>dos*<br>needle<br>device<br>syringe<br>pen<br>delivery	adheren*<br><br>nonadheren*<br>non-adheren*<br>non adheren*<br><br>complian*<br>noncomplian*<br>non-complian*<br>non complian*<br><br>persistence<br>nonpersisten*<br>non-persisten*<br>non persisten*<br><br>concordan*<br>discordan*<br><br>continu*<br>discontinu*<br>dis-continu*<br><br>duration<br>cessation	intervention<br>random*<br>non-random*<br>non random*<br>quasi*<br>clinical<br>control*<br>trial<br>experiment*<br>outcome

Supplementary Table S2 Main reasons for study exclusion table

Author	Study Title	Publication Year	Primary Reason for Exclusion
Amereller et al.[1]	Adherence, attitudes and beliefs of growth hormone deficient patients–a questionnaire-based cohort study	2021	• Not an interventional study designed to improve adherence.
Assefi et al.[2]	Positive Impact on Adherence Through Educational Activities of the Argentina´s Patient Support Program in Children with low Adherence to Treatment with Recombinant Growth Hormone (easypod applicator)	2019	• Full-text copy not available (only available in abstract form)
Assefi et al.[3]	Positive Impact of Targeted Educational Intervention in Children With Low Adherence to Growth Hormone Treatment Identified by Use of the Easypod™ Electronic Auto-injector Device	2021	• Not an interventional study designed to improve adherence.
Aydın et al.[4]	Adherence to growth hormone therapy: results of a multicenter study.	2014	• Not an interventional study designed to improve adherence.
Ayuk et al.[5]	Growth hormone device change-over; is it beneficial?	2013	• Full-text copy not available (only available in abstract form)
Bagnasco et al.[6]	Adherence Investigators Group*. Prevalence and correlates of adherence in children and adolescents treated with growth hormone: a multicenter Italian study.	2017	• Not an interventional study designed to improve adherence.
Bercu et al.[7]	Long-term therapy with recombinant human growth hormone (Saizen®) in children with idiopathic and organic growth hormone deficiency.	2001	• Not an interventional study designed to improve adherence.
Bozzola et al.[8]	Adherence to growth hormone therapy: a practical approach.	2014	• Not an interventional study designed to improve adherence.
Brearley et al.[9]	Pharmacokinetics of recombinant human growth hormone administered by cool. click™ 2, a new needle-free device, compared with subcutaneous administration using a conventional syringe and needle.	2007	• Adherence not assessed/measured

Choi[10]	Improving adherence to growth hormone (GH) therapy via Easypod^{TM} may help maximise the treatment outcome	2015	• Not an interventional study designed to improve adherence (review).
Coutant et al.[11]	Patients' perceptions on the usability of the SurePal™ self-injection device for Omnitrope®: a questionnaire-based observational study conducted in paediatric patients in France	2017	• Adherence not assessed/measured (acceptability study)
Cutfield et al.[12]	Final height in idiopathic growth hormone deficiency: the KIGS experience.	1999	• Adherence not assessed/measured
Cutfield et al.[13]	Non-compliance with growth hormone treatment in children is common and impairs linear growth.	2011	• Not an interventional study designed to improve adherence.
Dahlgren et al.[14]	Patient acceptance of a novel electronic auto-injector device to administer recombinant human growth hormone: results from an open-label, user survey of everyday use.	2007	• Adherence not assessed/measured (acceptability study)
Dahlgren et al.[15]	Easypod® a new electronic injection device for growth hormone.	2008	• Not an interventional study designed to improve adherence (review).
De Pedro et al.[16]	Variability in adherence to rhGH treatment: Socioeconomic causes and effect on children's growth.	2016	• Not an interventional study designed to improve adherence.
Dörr et al.[17]	Are needle-free injections a useful alternative for growth hormone therapy in children? Safety and pharmacokinetics of growth hormone delivered by a new needle-free injection device compared to a fine gauge needle.	2003	• Patient group includes >18 years. ≤18 data not extractable.
Dumas et al.[18]	Understanding and meeting the needs of those using growth hormone injection devices.	2006	• Not an interventional study designed to improve adherence (review).
Farfel et al.[19]	Long-term adherence to growth hormone therapy in a large health maintenance organization cohort.	2019	• Not an interventional study designed to improve adherence.
Fuchs et al.[20]	Ease of use and acceptability of a new pen device for the administration of growth hormone therapy in pediatric patients: an open-label, uncontrolled usability test.	2009	• Adherence not assessed/measured (acceptability study)
Graham et al.[21]	Exploring Potentially Modifiable Factors That Influence Treatment Non-Adherence Amongst Pediatric Growth Hormone Deficiency: A Qualitative Study	2020	• Not an interventional study designed to improve adherence.

Graham et al.[22]	What potentially modifiable factors are associated with treatment non-adherence in pediatric growth hormone deficiency? A quantitative study.	2021	• Not an interventional study designed to improve adherence.
Haverkamp et al.[23]	Observations of nonadherence to recombinant human growth hormone therapy in clinical practice.	2008	• Not an interventional study designed to improve adherence (review).
Hey-Hadavi et al.[24]	Ease of use and preference for a new disposable self-injection pen compared with a reusable pen for administering recombinant human growth hormone: A multicenter, 2-month, single-arm, open-label clinical trial in patient-caregiver dyads.	2010	• Adherence not assessed/measured (acceptability study)
Hindmarsh & Brook[25]	Compliance with growth hormone treatment – is it a problem?	1999	• Not an interventional study designed to improve adherence (review).
Hokken-Koelega et al.[26]	Patient acceptance, ease of use, and preference for Norditropin NordiFlex with NordiFlex PenMate: results from an open-label, user survey of everyday use.	2011	• Adherence not assessed/measured (acceptability study)
Johannsson[27]	Long-acting growth hormone for replacement therapy.	2011	• Not an interventional study designed to improve adherence (editorial review).
Kale et al.[28]	Needle free injection technology - An overview.	2014	• Not an interventional study designed to improve adherence (review).
Kapoor et al.[29]	Monitoring of concordance in growth hormone therapy.	2008	• Not an interventional study designed to improve adherence.
Kappelgaard et al.[30]	Patient preference for a new growth hormone injection device: results of an open-label study in Japanese pediatric patients.	2011	• Adherence not assessed/measured (acceptability study)
Kappelgaard et al.[31]	Children and adolescent acceptability of a new device system to administer human growth hormone–a pilot study.	2012	• Adherence not assessed/measured (acceptability study)
Kappelgaard & Hansen[32]	Comparison of injection dose force, accuracy and precision among three growth hormone injection devices.	2013	• Adherence not assessed/measured
Kappelgaard & Laursen[33]	The benefits of growth hormone therapy in patients with Turner syndrome, Noonan syndrome and children born small for gestational age.	2011	• Not an interventional study designed to improve adherence (review).

Kappelgaard et al.[34]	A web-based survey assessing the impact of storage flexibility on the daily life of patients and caregivers administering growth hormone.	2015	• Patient group includes >18 years. ≤18 data not extractable.
Kaptein et al.[35]	Transjecting growth hormone: continuous nightmare or controlled nuisance? Evaluation of a new needle-free device.	2013	• Adherence not assessed/measured (acceptability study)
Khadilkar et al.[36]	24-Month Use of Once-Weekly GH, LB03002, in Prepubertal Children With GH Deficiency	2014	• Adherence not assessed/measured
Koledova et al.[37]	Adherence and long-term growth outcomes: results from the easypod™ connect observational study (ECOS) in paediatric patients with growth disorders.	2018	• Patient group includes >18 years. ≤18 data not extractable.
Lass et al.[38]	Low treatment adherence in pubertal children treated with thyroxin or growth hormone.	2015	• Not an interventional study designed to improve adherence.
Lopez Siguero et al.[39]	Treatment with growth hormone: what do children know and how do they accept it?	1995	• Not an interventional study designed to improve adherence (review).
Luo X et al.[40]	Long-acting PEGylated recombinant human growth hormone (Jintrolong) for children with growth hormone deficiency: phase II and phase III multicenter, randomized studies	2017	• Clearly-defined measure of adherence not identified.
Lustig[41]	Optimizing growth hormone efficacy: an evidence-based analysis.	2004	• Not an interventional study designed to improve adherence (review).
Main et al.[42]	Automatic needle insertion diminishes pain during growth hormone injection.	1995	• Adherence not assessed/measured
Meinhardt et al.[43]	Parent preference in Switzerland for easy-to-use attributes of growth hormone injection devices quantified by willingness to pay.	2014	• Sample group: Participants were parents who had children *without* short stature
Miller et al.[44]	Persistence with growth hormone therapy in pediatric patients.	2014	• Not an interventional study designed to improve adherence.
Mitragotri[45]	Current status and future prospects of needle-free liquid jet injectors.	2006	• Current status and future prospects of needle-free liquid jet injectors.
Mohseni et al.[46]	Adherence to growth hormone therapy in children and its potential barriers.	2018	• Not an interventional study designed to improve adherence.

Moore et al.[47]	A Randomized Safety and Efficacy Study of Somavaratan (VRS-317), a Long-Acting rhGH, in Pediatric Growth Hormone Deficiency	2016	• Adherence not assessed/measured
Müller et al.[48]	Norditropin® SimpleXxTM: a liquid human growth hormone formulation, a pen system and an auto-insertion device.	1999	• Adherence not assessed/measured (acceptability study)
Nicolino et al.[49]	Easypod Connect Observational Study (ECOS) – French Case Histories and Growth Outcomes.	2017	• Full-text copy not available (only available in abstract form)
Norgren[50]	Adherence remains a challenge for patients receiving growth hormone therapy.	2009	• Not an interventional study designed to improve adherence (review).
Osterberg & Blaschke[51]	Adherence to medication.	2005	• Not an interventional study designed to improve adherence (review).
Oyarzabal et al.[52]	Multicentre survey on compliance with growth hormone therapy: what can be improved?	1998	• Not an interventional study designed to improve adherence (review).
Partsch et al.[53]	Acceptability of the reusable surePal™ self-injection device for Omnitrope® among pediatric patients: results from a questionnaire-based, cross-sectional, multicenter observational study.	2015	• Adherence not assessed/measured (acceptability study)
Pfützner et al.[54]	Intuitiveness, ease of use, and preference of a prefilled growth hormone injection pen: a noninterventional, randomized, open-label, crossover, comparative usability study of three delivery devices in growth hormone-treated pediatric patients.	2010	• Adherence not assessed/measured (acceptability study)
Pleil et al.[55]	Results from an international multicenter trial evaluating the ease-of-use of and preference for a newly developed disposable injection pen for the treatment of growth hormone deficiency in treatment-naïve children and adults.	2014	• Adherence not assessed/measured (acceptability study)
Rapaport et al.[56]	Validation and ease of use of a new pen device for self-administration of recombinant human growth hormone: results from a two-center usability study.	2013	• Adherence not assessed/measured (acceptability study)
Rosenfeld & Bakker[57]	Compliance and persistence in pediatric and adult patients receiving growth hormone therapy.	2008	• Not an interventional study designed to improve adherence.

Sauer & Abbotts[58]	A new pen device for injection of recombinant human growth hormone: a convenience, functionality and usability evaluation study.	2018	• Adherence not assessed/measured (acceptability study)
Shine et al.[59]	Patient and parent preference for growth hormone products.	2003	• Adherence not assessed/measured (letter)
Silverstein et al.[60]	Clinical testing results and high patient satisfaction with a new needle-free device for growth hormone in young children.	2001	• Sample group: Participants had type 1 diabetes mellitus (i.e. did not have clinical diagnosis of short stature or growth failure approved by the FDA)
Sjöblom et al.[61]	Patient evaluation of a new injection pen for growth hormone treatment in children and adults.	1995	• Adherence not assessed/measured (acceptability study)
Smith et al.[62]	Compliance with growth hormone treatment – are they getting it?	1993	• Not an interventional study designed to improve adherence (review).
Stanhope et al.[63]	Optimum method for administration of biosynthetic human growth hormone: a randomised crossover trial of an Auto Injector and a pen injection system.	1992	• Adherence not assessed/measured
Stanhope et al.[64]	Patient knowledge and compliance with growth hormone treatment.	1993	• Not an interventional study designed to improve adherence (review).
Staples & Bravender[65]	Drug compliance in adolescents: assessing and managing modifiable risk factors.	2002	• Not an interventional study designed to improve adherence (review).
Tauber et al.[66]	User trial of Easypod®, an electronic autoinjector for growth hormone.	2008	• Adherence not assessed/measured (acceptability study)
Tebbi[67]	Treatment compliance in childhood and adolescence.	1993	• Not an interventional study designed to improve adherence (review).
Tollerfield[68]	Facilitating the adherence journey of children, adolescents and adults on long-term growth hormone therapy	2020	• Not an interventional study designed to improve adherence (review).
Verrips et al.[69]	Psychological responses to the needle-free Medi-Jector® or the multidose Disetronic® injection pen in human growth hormone therapy.	1998	• Patient group includes >18 years. ≤18 data not extractable.

Weill & Niez[70]	Adherence to the treatment with Zomajet, a needle-free device transjecting growth hormone: results of French observational survey.	2013	• Full-text copy not available (only available in poster form)
Zamora Ferrer[71]	Pharmacotherapeutic monitoring in growth-hormone treatment adherence.	2015	• Full-text copy not available (only available in abstract form)

Supplementary Table 3 Full data extraction table

a) Study details and participant characteristics

**Electronic auto-injector**									
**Study details**				**Participant characteristics**					
**Author**	**Publication Year**	**Country**	**Study Design**	**Sample Size**	**Sample Groupings**	**Gender N (%)**	**Mean Age: y.m**	**Clinical Indication of GH Therapy N (%)**	**Duration of GH Therapy**
Arrabal Vela et al.[72]	(2018)	Spain	Retrospective, longitudinal descriptive study	30 pediatric patients	-	M = 17 (56.6%)<br>F = 13 (43.3%)	Total mean age = 6.09 (4.92-7.25) years	SGA = 17 (56.6%)<br>GHD = 11 (36.6%)<br>TS = 2 (6.7%)	Mean = 4.3 (range = 3.6-5) years
Blanco-López et al.[73]	(2020)	Mexico	National, multicentre, longitudinal observational study<br><br>*[Easypod Connect Observational Study]*	147 pediatric patients	Treatment naïve (71.4%)]<br>Treatment established (28.6%)	M = 83 (56.5%)<br>F = 64 (43.5%)	Total mean age = 9.96 ± 3.41 years	GHD = 118 (80.3%)<br>SGA = 24 (16.3%)<br>TS = 5 (3.4%)	Not reported
Bozzola et al.[74]	(2011)	Inter-national (*n*=15)	Multicentre, multinational, observational survey study	824 pediatric patients	rhGH treatment-naïve = 601 (72.9%)<br>rhGH treatment-experienced = 223 (27.1%)	M = 462 (56.1%)<br>F = 362 (43.9%)	Total median age (range) = 11 (1-18) years	GHD = 543 (65.9%)<br>TS = 80 (9.7%)<br>SGA = 125 (15.2%)<br>Chronic renal failure (CRF) = 14 (1.7%)<br>Other = 56 (6.8%)	Not reported
Centonze et al.[75]	(2019)	Italy	Prospective, longitudinal, observational study	73 treatment-naïve pediatric patients	-	M = 38 (52.1%)<br>F = 35 (47.9%)	Total mean age = 9.78 ± 3.20 years	Idiopathic GHD = 70 (95.9%)<br>Organic GHD = 2 (2.7%)	Not reported

			[Easypod Connect Observational Study]					Congenital GHD = 1 (1.4%)	
Hartmann et al.[76]	(2013)	Germany	Prospective observational study	75 pediatric patients	Prepubertal = 29 (38.7%); Pubertal = 41 (54.7%)**	M = 46 (61.3%) F = 29 (38.7%)	Total mean age = 12.5 ± 3.5 years	GHD = 48 (64.0%) SGA = 18 (24.0%) TS = 6 (8.0%) CRF = 3 (4.0%)	Not reported
Loche et al.[77]	(2016)	Italy	Prospective observational study	79 pediatric patients	-	M =52 (65.8%) F = 27 (34.2%)	Median age at enrolment (interquartile range) = 10 (9-12) years	GHD = 100%	Not reported.
Maggio et al.[78]	(2018)	Italy	Retrospective, observational monocentric study	40 pediatric patients	Prepubertal = 30 (75%); Pubertal = 10 (15%)	M = 27 (67.5%) F = 13 (32.5%)	Total mean age = 11.2 ± 2.3 years	Isolated GHD = 26 (65%) SGA = 9 (22.5%) TS = 5 (12.5%)	2.41 ± 1.86 years
Rodríguez Arnao et al.[79]	(2019)	Spain	National, multicentre, prospective observational study. *[Easypod Connect Observational Study]*	238 pediatric patients	Prepubertal = 212 (89.1%) Pubertal = 26 (10.9%)	M = 123 (51.7%) F = 115 (48.3%)	Total mean age at inclusion (±SD) = 9.0 ± 3.3 years Total mean age at treatment initiation = 7.9 ± 3.2 years	Growth hormone deficiency (GHD) = 144 (60.5%) Small for gestational age (SGA) = 86 (36.1%) Turner syndrome (TS) = 8 (3.4%)	Mean = 26.6 ± 11.6 months
van Dommelen et al.[80]	(2018)	Nether-lands	Prospective observational study *[Easypod Connect Observational Study]*	95 treatment-naïve pediatric patients	-	M = 72 (75.8%) F = 23 (24.2%)	Mean age = 6.3 ± 2.1 years	Idiopathic isolated GHD = 100%	Not reported
**Needle-free injector**									

Study details				Participant characteristics					
**Author**	**Publication Year**	**Country**	**Study Design**	**Sample Size**	**Sample Groupings**	**Gender N (%)**	**Mean Age: y.m**	**Clinical Indication of GH Therapy N (%)**	**Duration of GH Therapy**
Desrosiers et al.[81]	(2005)	USA	Retrospective cohort study	631 pediatric patients	Needle and syringe = 305 (48.3%);<br><br>Cool.click needle-free device = 326 (51.7%)	*NFDS Patients:*<br>M = 222 (68.1%)<br>F = 104 (31.9%)<br><br>*Needle Device patients:*<br>M = 204 (66.9%)<br>F = 101 (33.1%)	*NFDS Patients:*<br>Total mean age: 10.6 ± 3.9<br><br>*Needle Device patients:*<br>Total mean age:<br>10.1 ± 3.9	*NFDS Patients:*<br>Idiopathic GH deficiency =218 (78.7%)<br>Turner syndrome = 16 (5.8%)<br>Organic GH deficiency = 7 (2.5%)<br>Other dysmorphic 21 (7.6%)<br>Small for gestational age = 6 (2.2%)<br>Prader-Willi syndrome 2 (0.7%)<br>Neurosecretory dysfunction 4 (1.4%)<br>Noonan syndrome = 1 (0.4%)<br>Crondrodystrophy = 1 (0.4%)<br>Chronic kidney disease = 0 (0.0%)<br>Congenital adrenal hyperplasia = 1 (0.4%)<br>Genetic GH deficiency = 0 (0.0)<br>Hypophosphatemic rickets 0 (0.0)<br><br>*Needle Device patients:*<br>Idiopathic GH deficiency = 164 (72.9%)<br>Turner syndrome = 16 (7.1%)<br>Organic GH deficiency = 19 (8.4%)<br>Other dymorphic = 3 (1.3%)<br>Small for gestational age = 7 (3.1%)<br>Prader-Willi syndrome 7 (3.1%)	Not reported

								Neurosecretory dysfunction = 3 (1.3%)<br>Noonan syndrome = = 3 (1.3%)<br>Crondrodystrophy = 0 (0.0%)<br>Chronic kidney disease = 1 (0.4%)<br>Congenital adrenal hyperplasia = 0 (0.0%)<br>Genetic GH deficiency = 1 (0.4)<br>Hypophosphatemic rickets 1 (0.4)	
Michaelidou et al.[82]	(2019)	UK	Retrospective longitudinal study	*1-year treatment cohort:*<br>52 pediatric patients<br><br>*3-year treatment cohort:*<br>22 pediatric patients	-	*1-year treatment cohort:*<br>M = 30 (57.7%)<br>F = 22 (42.3%)<br>*3-year treatment cohort:*<br>M = 11 (50.0%)<br>F = 11 (50.0%)	*1-year treatment cohort:*<br>Total mean age = 8.50 ± 3.78 years<br><br>*3-year treatment cohort:*<br>Total mean age = 7.21 ± 3.68 years	*1-year treatment cohort:*<br>GHD = 34 (65.4%)<br>TS = 5 (9.6%)<br>Other = 13 (25.0%)<br><br>*3-year treatment cohort:*<br>GHD = 17 (77.3%)<br>TS = 2 (9.1%)<br>Other = 3 (13.6%)	Not reported
Spoudeas et al.[83]	(2014)	UK	Retrospective observational study	4,093 pediatric patients<br><br>*NB:* Adherence was examined in patients using ZomaJet = 728 (17.8%).	Zomacton via the ZomaJet device = 728 (17.8%)<br>Needle-based devices = 3,365 (82.2%)	*ZomaJet device:*<br>M = 423 (58.1%)<br>F = 304 (41.8%)<br>[Gender not specified for 1 patient)<br><br>*Needle-based devices:*<br>M = 1,931 (57.4%)<br>F = 1,493 (44.4%)<br>[Gender not specified for 3 patients]	*ZomaJet device:*<br>Total mean age = 8.4 ± 4.0 years<br><br>*Needle-based devices:*<br>Total mean age = 9.7 ± 4.3 years	*ZomaJet device:*<br>Mixed conditions treated with rhGH<br><br>*Needle-based devices:*<br>Mixed conditions treated with rhGH	Not reported

**Injector pen**									
**Study details**				**Participant characteristics**					
**Author**	**Publication Year**	**Country**	**Study Design**	**Sample Size**	**Sample Groupings**	**Gender N (%)**	**Mean Age: y.m**	**Clinical Indication of GH Therapy N (%)**	**Duration of GH Therapy**
Tauber et al.[84]	(2013)	France	Prospective, multicentre, open-label study	103 pediatric patients	-	M = 60 (58.3%)<br>F = 43 (41.7%)	Total mean age = 11.7 ± 2.9 years	SGA = 51 (49.5%)<br>GHD = 43 (41.7%)<br>TS = 9 (8.7%)	Median = 3.6 (range: 0.5-14.3) years

**Patient Choice**									
**Study details**				**Participant characteristics**					
**Author**	**Publication Year**	**Country**	**Study Design**	**Sample Size**	**Sample Groupings**	**Gender N (%)**	**Mean Age: y.m**	**Clinical Indication of GH Therapy. N (%)**	**Duration of GH Therapy**
Gau & Takasawa[85]	(2017)	Japan	Retrospective, longitudinal survey study	46 pediatric patients	Non-patient choice group *n* = 18 (39.1%);<br>- All patient choice group *n* = 28 (60.9%)	M = 24 (52.2%)<br>F = 22 (47.8%)	Mean age = 7.70 ± 3.12 years	Isolated and idiopathic GHD = 100%	Not reported
Wickramasuriya et al.[86]	(2006)	UK	Prospective cross-sectional study	125 treatment-naïve pediatric patients<br><br>*NB:* Compliance assessed in 50 (40%) children who received GH by hospital prescription	8 (6%) children under the age of 2 years.<br>33 (26%) pre-school children	M = 74 (59.2%)<br>F = 51 (40.8%)	Median age (range) = 9.30 (1.0-18.3) years	GHI = 69 (55%) [of which 29 were post-oncology and 4 with organic GHI due to midline defects (septo-optic dyplasia)]<br>Turner Syndrome = 16 (13%)<br>Small for gestational age 10 (8%)<br>Chronic renal insufficiency = 8 (7%)<br>Prader-Willi syndrome = 3 (2%)<br>Others = 19 (15%)	Commencing GH therapy<br><br>50 (40%) have had hospital tracking/ prescription and home delivery of GH treatment for >1 year

				and home delivery				[Skeletal dysplasia = 4<br>Rhematology = 3<br>Extreme short stature = 3,<br>undiagnosed dysmorphic syndrome = 3<br>Noonan syndrome = 1<br>VATER syndrome = 1<br>Holt-Oram syndrome = 1<br>Cushing disease =1<br>osteogenesis imperfecta = 1;<br>and short gut syndrome = 1]	

b) Study details and intervention features

**Electronic auto-injector**				
**Study details**			**Intervention Features**	
**Author**	**Study objectives**	**Adherence as an outcome**	**Intervention**	**Control Group**
Arrabal Vela et al.[72]	**1.** Monitor adherence in pediatric subjects receiving rhGH via the easypod device.<br><br>**2.** Determine the relationship between adherence and the following variables: age; gender; treatment duration; diagnosis; degree of height Impairment; and growth rate.	Primary	easypod® device (Merck Serono S.A., Geneva, Switzerland) - a hidden-needle auto-injector	Within-subject pre-post test
Blanco-López et al.[73]	**1.** Assess the level of adherence in pediatric subjects receiving rhGH via the easypod device over a follow-up period of 3 years.<br><br>**2.** Assess the impact of adherence on the following growth outcomes: height velocity; height velocity SDS; height; height SDS; and IGF-1 concentrations.	Primary	easypod® device (Merck Serono S.A., Geneva, Switzerland) - a hidden-needle auto-injector	Within-subject pre-post test
Bozzola et al.[74]	**1.** Assess adherence in treatment-naïve and treatment-experienced pediatric patients receiving rhGH via the easypod device.<br><br>**2.** Assess the acceptability of the easypod device.	Primary	easypod® device (Merck Serono S.A., Geneva, Switzerland) - a hidden-needle auto-injector	Within-subject pre-post test
Centonze et al.[75]	**1.** Assess the level of adherence in treatment-naïve pediatric subjects receiving rhGH via the easypod device over a follow-up period of 3 years.	Primary	easypod® device (Merck Serono S.A., Geneva, Switzerland) - a hidden-needle auto-injector	Within-subject pre-post test
Hartmann et al.[76]	**1.** Assess the level of adherence in pediatric subjects receiving rhGH via the easypod device.	Primary	easypod® device (Merck Serono S.A., Geneva, Switzerland) - a hidden-needle auto-injector	Within-subject pre-post test

Loche et al.[77]	**1.** Monitor adherence in pediatric GHD subjects receiving rhGH via the easypod device over a 1-year period.<br><br>**2.** Monitor the effect of rhGH treatment on serum IGF-1 concentrations, fasting serum glucose and insulin, and on anthropometric characteristics (height, waist circumference, and BMI).	Primary	easypod® device (Merck Serono S.A., Geneva, Switzerland) - a hidden-needle auto-injector	Within-subject pre-post test
Maggio et al.[78]	**1.** Monitor adherence in pediatric subjects receiving rhGH via the easypod device.<br><br>**2.** (i) Study rhGH efficacy in growth improvement and height gain, considering both efficacy and treatment adherence; (ii) limit waste involved in expensive therapies for the national health system; (ii) optimize GH therapy, improving the quality of patient assistance.	Primary	easypod® device (Merck Serono S.A., Geneva, Switzerland) - a hidden-needle auto-injector	Within-subject pre-post test
Rodríguez Arnao et al.[79]	**1.** Assess the level of adherence in pediatric subjects receiving recombinant human growth hormone (rhGH) via the easypod device over a follow-up period of 4 years.<br><br>**2.** i) Assess the impact of adherence on growth outcomes; ii) identification of demographic and auxological characteristics of subjects associated with adherence; and iii) assessment of the impact of insulin-like growth factor I (IGF-1) levels on adherence over the follow-up period.	Primary	easypod® device (Merck Serono S.A., Geneva, Switzerland) - a hidden-needle auto-injector	Within-subject pre-post test
van Dommelen et al.[80]	**1.** Investigate the effect of adherence on the 2-year growth response to growth hormone treatment in prepubertal children with idiopathic isolated GHD.	Primary	easypod® device (Merck Serono S.A., Geneva, Switzerland) - a hidden-needle auto-injector	Within-subject pre-post test

**Needle-free injector**				
**Study details**			**Intervention Features**	
**Author**	**Study objectives**	**Adherence as an outcome**	**Intervention**	**Control Group**

Desrosiers et al.[81]	**1.** Examine the impact of rhGH administration method (needle and syringe vs cool.click needle-free device) on growth outcomes (growth rate, change in height SDS, change in height age).<br><br>**2.** Examine the impact of rhGH administration method on treatment compliance.	Secondary	Cool.click™ device (Merck Serono International S.A., Geneva, Switzerland) - a needle-free device	Needle and syringe injection group
Michaelidou et al.[82]	**1.** Assess the effect of adherence on medium-term (3 years) growth outcomes in terms of height standard deviation scores (HTSDS).<br><br>**2.** Assess the difference from target mid-parental height SDS (MPHSDS – HTSDS) in children receiving rhGH via ZomaJet.	Primary	ZomaJet® device (Ferring Pharmaceuticals, London, UK) - a needle-free device	Within-subject pre-post test
Spoudeas et al.[83]	**1.** Investigate how the use of a needle-free impacted device (ZomaJet) impacts persistence and adherence compared to needle-based devices.	Primary	ZomaJet® device (Ferring Pharmaceuticals, London, UK) - a needle-free device	Within-subject pre-post test [*Adherence*]<br>*NB:* Adherence was only examined in patients using ZomaJet.<br><br>Needle-based device group [*Persistence*]

**Injector pen**				
**Study details**			**Intervention Features**	
**Author**	**Study objectives**	**Adherence as an outcome**	**Intervention**	**Control Group**
Tauber et al.[84]	**1**. Assess the ease of use of NordiFlex compared with that of other growth hormone injection devices previously used to treat children with rhGH.<br><br>**2.** (i) Assess the ease of learning how to administer NordiFlex, (ii) patient autonomy assessment, (iii) patient or parent preference for GH injection device, (iv) adherence, and (v) the safety of NordiFlex.	Secondary	NordiFlex® device (Novo Nordisk A/S, Bagsvaerd, Denmark) - a prefilled, multidose, disposable injector pen	Within-subject pre-post test

Patient Choice				
**Study details**			**Intervention Features**	
**Author**	**Study objectives**	**Adherence as an outcome**	**Intervention**	**Control Group**
Gau & Takasawa[85]	**1.** Assess the impact of patient choice of a GH device on adherence to and therapeutic effects of rhGH over a 3-year period.	Primary	Patient choice of an injection device	Non-patient choice group [Treatment as Usual]
Wickramasuriya et al.[86]	**1.** Investigate the factors determining patient choice of GH device.<br><br>**2.** Investigate whether offering patient choice improved rhGH compliance.	Secondary	Patient choice of an injection device	Non-patient choice group

c) Study details and adherence measurement

Electronic auto-injector					
**Study details**	**Adherence Measurement**				
**Author**	**Reported by whom**	**Adherence measure**	**Assessment calculation**	**Observation Time Period**	**Definition of non-adherence**
Arrabal Vela et al.[72]	-	Electronic monitoring device - easypod® device	Adherence was calculated as the percentage of injections received (*days*) with respect to planned injections.	12-months	Adherence was categorized as follows: excellent adherence (>95%); good adherence (95-85%); fair adherence (85-75%); and poor adherence (<75%).
Blanco-López et al.[73]	-	Electronic monitoring device - easypod® device	Adherence was calculated as the percentage of injections received (*days*) with respect to planned injections.	3-month, 6-month, 1-year, 2 years, 3 years	Not reported
Bozzola et al.[74]	Patient or parents	Electronic monitoring device (*recorded adherence*) in conjunction with a patient/parent self-report survey (*reported adherence*)	*Recorded* adherence (via easypod) was calculated by the imputation of missing period(s) using non-missing period(s).<br><br>*Reported* adherence (via survey) was calculated by the number of missed injections (e.g. 0; 1-3; 4-6; 7-9; ≥10).	3-months	Adherence was defined as those with ≥92% adherence to prescribed treatment (no more than two daily injections missed per month or six daily injections for the 3-month period).
Centonze et al.[75]	-	Electronic monitoring device (easypod®)	Adherence was calculated as the percentage of injections recorded versus prescribed.	1 years, 2 years, 3 years	Not reported
Hartmann et al.[76]	-	Electronic monitoring device (easypod®) in conjunction with a clinical kit software	Adherence was assessed with respect to the proportion of actual injected rhGH compared with the prescribed dose.	The average observation time was 343 ± 201 days<br><br>(range 28–1,034 days)	Adherence was categorized using Cutfield et al.'s[13] definitions of compliance: good compliance = fewer than 1 missed dose per week (85.7–100% proportion injected); medium compliance = 1–3

					missed doses per week (57.1–85.7% proportion injected); and poor compliance = more than 3 missed doses per week (<57.1% proportion injected).
Loche et al.[77]	-	Electronic monitoring device (easypod®)	Adherence was calculated as the percentage of injections received (*days*) with respect to planned injections.	1 year	The adherence population was defined as those with ≥92% adherence to prescribed treatment.
Maggio et al.[78]	Patient and parents	Electronic monitoring device (*recorded adherence*) in conjunction with a patient/parent self-report survey (*reported adherence*)	*Recorded* adherence (via easypod) was calculated as the percentage of injections received (*days*) with respect to planned injections.	Data were collected at baseline, (before the treatment start), and after appropriate follow-up, which was variable for each patient, according to clinical practice.	Not reported
Rodríguez Arnao et al.[79]	-	Electronic monitoring device - easypod® device	Adherence was calculated as the percentage of injections received (*days*) with respect to planned injections.	6 months, 1 year, 2 years, 3 years and 4 years.	Adherence was defined as those with ≥85% adherence to prescribed treatment (no more than 1 missed dose a week on average).
van Dommelen et al.[80]	-	Electronic monitoring device (easypod® device) combined with physician data entry of outcome measures	Adherence was calculated as the percentage of injections received (*days*) with respect to planned injections.	2 years	Adherence was defined differently for each time-point: Year 1 = ≥98% adherence to treatment; Year 2 = ≥91%; First two years = ≥78%.

**Needle-free injector**

Study details	Adherence Assessment				
**Author**	**Reported by whom**	**Adherence measure**	**Assessment calculation**	**Observation Time Period**	**Definition of non-adherence**
Desrosiers et al.[81]	Physician	Physician report<br><br>*NB:* Adherence was reported for 609 of the 631 patients	Not reported	24 months	Adherence = defined as those who missed <3 doses per month.
Michaelidou et al.[82]	-	Issued, renewed, or redeemed rhGH prescriptions	Proportion of days covered (PDC) = Number of days with access to viable heads/number of days receiving treatment.	3 years	PDC score >0.8 = Highly adherent.
Spoudeas et al.[83]	-	Issued, renewed, or redeemed rhGH prescriptions<br><br>*NB:* Adherence was only examined in patients using ZomaJet.	*Adherence:* PDC = Number of days with access to viable heads/number of days receiving treatment.<br><br>*Persistence:* Time interval between a patient's first and last delivery of GH for each GH brand.	3 years	PDC score >0.8 = Highly adherent.

Injector pen

Study details	Adherence Assessment				
**Author**	**Reported by whom**	**Adherence measure**	**Assessment calculation**	**Observation Time Period**	**Definition of non-adherence**
Tauber et al.[84]	Patient and/or parents	Used patient/parent diaries	Not reported	6-weeks	The "absolutely adherent" population was defined as those who missed no daily rhGH dose during the 6-week study period.

Patient Choice					
**Study details**	**Adherence Assessment**				
**Author**	**Reported by whom**	**Adherence measure**	**Assessment calculation**	**Observation Time Period**	**Definition of non-adherence**
Gau & Takasawa[85]	Patient and/or Parent/care-giver	Self-report questionnaires<br><br>Auxological and hormonal data over a 3-year period were used to assess the impact of patient choice.	Not reported	3 years	Not reported
Wickramasuriya et al.[86]	-	Ampoule counts	Comparing the number of ampoules of GH used against expected ampoule usage. Percentage compliance for each patient was produced by dividing the actual number of GH ampoules used by the expected GH ampoule use x 100%	3 years	Not reported

d) Study details and key findings

Electronic auto-injector		
**Study details**	**Key findings**	
**Author**	**Adherence/ Non-adherence rate**	**Further findings**
Arrabal Vela et al.[72]	Mean treatment adherence was 92.3%.<br><br>According to the adherence categories:<br>60% of the patients were defined as excellent compliers,<br>30% good compliers<br>3.3% fair and<br>6.7% poor compliers	*Adherence and aforementioned variables:*<br>A significant negative correlation was observed between adherence and age (r = -0.384, p = 0.030) and treatment duration (r = -0.537, p = 0.003).<br><br>No significant relationship was discovered between adherence and initial height (r = -0.143, p = 0.452) and final height (r = 0.143, p = 0.460), or between adherence and growth rate (r = 0.136, p = 0.481).<br><br>There were no differences in adherence between males and females (p = 0.815) and treatment indication (p = 0.085).
Blanco-López et al.[73]	Mean adherence was: >80%<br><br>90.4% (n = 146) at the 3-month follow-up<br>87.4% (n = 143) at the 6-month follow-up<br>85.7% (n = 135) at the 1-year follow-up<br>83.9% (n = 97) at the 2-year follow-up<br>84.5% (n = 39) at the 3-year follow-up	*Adherence:* Adherence was not significantly different by rhGH indication, nor between growth hormone naïve or experienced patients over the 3-year follow-up period.<br><br>*Adherence and growth outcomes:* [Due to the high attrition rate, the association of adherence with growth outcomes was only reported for the first year of treatment (n = 147)].<br>A statistically significant correlation was observed between treatment adherence and change in height SDS (r = 0.239, p = 0.005), in addition to height velocity SDS (r = 0.194, p = 0.027).
Bozzola et al.[74]	*Recorded adherence:* According to the recorded adherence data, 87.5% of children were adherent to treatment over the 3-month period.<br><br>Month 1 = 90.5%<br>Month 2 = 87.1%<br>Month 3 = 88.9%<br><br>51.4% (397/772) of children were recorded to have missed one or more injections over the 3-month period.<br><br>*Reported adherence:* According to self-reported data, 90.2% (n = 607/673) of children were adherent over 3 months; 51.5% (n = 421/817) missed ≥1 injection over this period.	*Adherence:* Recorded adherence was significantly greater in the treatment-naïve cohort (89.7%, n = 445/496) compared to the treatment-experienced cohort (81.7%, n = 152/186) [Fisher's exact test FI(X) = 7.577; p = 0.0062].<br><br>*Recorded Adherence* decreased with increasing treatment duration. In month 1, 75.1% (535/712) of children were recorded as missing no injections, in month 2, 66.7% (481/721), and in month 3, 66.7% (480/720). Over the same time period, the proportion of children missing 1-3 injections per month also increased (from 18.5% [132/712] to 24.4% [176/720]). A very small proportion of children missed >10 injections: only 3.1% (22/720) in month 3.<br><br>*Recorded vs reported adherence:* Reported adherence was higher (90.2%) compared to recorded adherence (87.5%) at each time point as well as overall.

Centonze et al.[75]	Mean adherence was >85% over the 3-year follow-up period:<br><br>1-year follow up = 88.5% (*n* = 65)<br>2-year follow-up = 86.6% (*n* = 40)<br>3-year follow-up = 86.5% (*n* = 18)	*Adherence:* mean adherence for individual treatment period (from treatment initiation to the last complete week of available data for each patient) was 86.5%. (*n* = 70).<br><br>After the first year of treatment, there was no significant correlation between the level of adherence and growth outcome.
Hartmann et al.[76]	The mean (±SD) rhGH treatment adherence rate of all patients was 91.2 ± 12.2%.<br><br>According to the definitions of Cutfield et al.,[13] 2.7% of all patients had poor compliance, 18.7% had medium compliance, and 78.7% had good compliance.<br><br>77.1% of patients with GHD showed good compliance. Approximately 90.0% of SGA patients were categorized as good compliers (10.0% medium, 10.0% poor). Approximately 50.0% of TS patients showed good compliance, while the remaining 50% were categorized as medium compliers. Approximately 100% of CRF patients showed good compliance.	*Adherence:* Adherence rate was observed in males (90.5 ± 13.1%) and females (92.2 ± 10.7%).<br><br>Compared to pubertal children (89.1 ± 13.7%), prepubertal children had a statistically significant higher mean adherence rate (96.5 ± 3.9%, $p < 0.005$).<br><br>There were no observable differences in mean and median adherence rates with respect to each rhGH indication: GHD (91.4 ± 11.0%, 95.5%); SGA (91.1 ± 15.3%, 96.0%); TS (86.0 ± 14.5%, 87.8%); CRF (99.3 ± 1.0%, 99.6%).<br><br>According to the definitions of Cutfield et al.,[13] adherence was similar between males and females.<br><br>In contrast, pubertal status and rhGH indication yielded some differences. 96.6% of prepubertal children were categorized as good and 3.4% as medium compliers. 70.7% of pubertal children were good compliers (24.4% medium, 4.9% poor).
Loche et al.[77]	The easypod™ data showed that 56.7 % of the patients were considered to be fully (≥92 %) adherent to their treatment throughout the 1-year study period.	*Adherence, height SDS, and IGF-1 SDS:* No significant correlation was found between the change in height SDS among fully adherent patients (≥300 injections across the 12-month follow-up period) and the adherence rate (coefficient β = 0.01241, $p = 0.123$).<br><br>Additionally, no significant correlation was observed between the changes observed in IGF-1 SDS and the adherence rate of patients with a minimum of 300 injections in the 12-month period (coefficient β = 0.01122, $p = 0.8517$).
Maggio et al.[78]	*Recorded adherence:* The mean treatment adherence was 92.20%.<br><br>1-year (96.0%, *n* = 13)<br>2–4 years (94.7%, *n* = 17)<br>4 years (83.9%, *n* = 10)<br><br>[Questionnaire Evaluation] *Reported adherence:*<br>Comparing the electronic evaluation of adherence, with the questionnaire answers, 26 patients (65.0%) referred a lower number of skip doses compared to what registered by easypod™,	*Recorded adherence:* mean treatment adherence (92.20%) was inversely related to patients' age ($r = -0.358$, $p = 0.023$), and significantly higher in the sub-group of patients with age between 10 and 13 years (96.43%, $p < 0.001$).<br><br>Treatment adherence showed an inverse correlation with the years of therapy ($r = -0.453$, $p = 0.003$) and with the number of rhGH administrations ($r = -0.392$, $p = 0.012$).<br><br>*Adherence and growth outcomes:* A significant correlation was yielded between treatment adherence and IGF-1 serum levels ($r = -0.398$, $p = 0.032$). However, no correlation was yielded between treatment adherence and final height ($r = 0.184$, *p*

	on the contrary 5 patients (12.5%) referred a higher number. Thus, 9 patients (22.5%) referred a skip doses number equal to what registered by the electronic device. In general, the mean skip doses number referred to parents was 1.3 doses monthly, although increasing until 2.5 doses monthly considering easypod™ data.	= 0.340) and growth velocity (*r* = 0.161, *p* = 0.422). Growth velocity was directly related to treatment adherence (*r* = 0.325, *p* = 0.044).
Rodríguez Arnao et al.[79]	Mean overall adherence was 94.5%.<br><br>Adherence was higher than 90% in all follow-up visits:<br>97.5% after 6 months [*n* = 234]<br>95.3% after 1-year [*n* = 232]<br>93.7% after 2 years [*n* = 174]<br>94.4% after 3 years [*n* = 84] and<br>95.5% after 4 years of treatment [*n* = 25]	*Adherence:* prepubertal and pubertal cohorts: 94.4% and 95.5% respectively. GHD and SGA cohorts, adherence was also similar at 95.2% and 93.0% respectively. No differences in adherence were observed between prepubertal and pubertal groups and GHD and SGA groups.<br><br>*Adherence and growth outcomes:*<br>Adherence significantly correlated with the following growth outcome variables:<br>Change in height after 1 (*r* = 0.170, *p* = 0.010) and 2 years (*r* = 0.217, *p* = 0.004), change in height standard deviation score (SDS) after 1 (*r* = 0.161, *p* = 0.015) and 2 years (*r* = 0.160, *p* = 0.035),<br>height velocity after 1-year (*r* = 0.206, *p* = 0.002),<br>height velocity SDS after at 1 (*r* = 0.168, *p* = 0.011) and 4 years (*r* = -0.473, *p* = 0.041),<br>change in body mass index (BMI) after 1-year (*r* = -0.193, *p* = 0.003) and<br>change in BMI SDS at 1 (*r* = -0.126, *p* = 0.002) and 2 years (*r* = -0.171, *p* = 0.051).<br><br>No significant difference in adherence according to IGF-1 levels was found in follow-up visits or between groups.
van Dommelen et al.[80]	In the first year:<br>32 children (34%) had a high adherence and<br>63 children (66%) a low adherence<br><br>In the second year:<br>50 children (53%) had high adherence and<br>45 children (47%) had low adherence<br><br>For the first two years:<br>68 children (72%) had high adherence, whilst<br>27 children (28%) had low adherence	*Adherence and growth outcomes:* The strongest associations were found between high adherence in the second year and index of responsiveness 2 years (+0.62); adherence and high adherence in the first two years and height SDS 0-2y (+0.11 SD per 1 injection/week, and +0.34 SD for high vs. low adherence).

**Needle-free injector**

Study details	Key findings	
**Author**	**Adherence/ Non-adherence rate**	**Further findings**
Desrosiers et al.[81]	Compliance was high in both the cool.click needle-free delivery system (84.6%) and needle and syringe (76.3%) cohort [missed <3 doses per month].<br><br>Compared to patients using the cool.click needle-free device, significantly more patients using needle and syringe missed over one-half of their prescribed GH dose (6% vs 13.4%, respectively, $p = 0.002$).	*Adherence and growth outcomes:* Compared to patients who missed fewer doses, growth outcomes were significantly lower in the group of poorly compliant patients.<br><br>Of the patients with compliance reports, 9.5% (58/609) were reported to have missed more than half (<15) of their monthly injections. The 12-month height velocity data in patients missing >15 injections per month (6.3/yr) was only 67% of the height velocity achieved by patients missing 11 to 15 doses (9.4cm/yr) ($p \leq .03$). Missing >15 doses was reported for half as many NFDS users as users of needle device (p=.01). The patients who missed >15 doses per month included 6% of the NFDS users versus 13.4% of the needle device users
Michaelidou et al.[82]	According to the 1-year data, 30 of the 52 patients (57.7%) were classified as adherent, whilst the remaining 22 patients (42.3%) were classified as less adherent.<br><br>According to the 3-year data, 14 of the 22 patients (63.6%) were classified as adherent, whilst the remaining 8 patients (36.4%) were classified as less adherent.	*HTSDS:* After 1-year of rhGH treatment, HTSDS was not significantly different in either adherence group.<br>After 3 years, only adherent patients exhibited sustained year-on-year increments in HTSDS and significant improvements in target HTSDS positions (by 1.32 SDS) compared to baseline ($p = 0.0008$).<br><br>*MPHSDS – HTSDS:* MPHSDS – HTSDS demonstrated significant improvements at 3 years in adherent patients only ($p = 0.0043$).
Spoudeas et al.[83]	*Adherence:* 424 of 728 ZomaJet using patients (58%) were classified as adherent (PDC 0.8–1.8).<br>Additionally, 175 of the 424 adherent patients (24%) were classified as over adherent (PDC > 1.8).<br><br>*Persistence:* Mean persistence was significantly longer in patients using ZomaJet than patients using needle-based devices (599 days vs 535 days, respectively; $p < 0.001$).	

Injector pen		
**Study details**	**Key findings**	
**Author**	**Adherence/ Non-adherence rate**	**Further findings**
Tauber et al.[84]	After the 6-week study period, 65/92 patients (70.6%) were classified as “absolutely adherent”.<br>Additionally, 13/92 patients (14.1%) had skipped only one GH injection during the 6-week period.	When questioned about the added value of NordiFlex, 27.2% of health care professionals suggested a positive impact on adherence.

Patient Choice		
**Study details**	**Key findings**	
**Author**	**Adherence/ Non-adherence rate**	**Further findings**
Gau & Takasawa[85]	Over the 3-year period, the non-patient choice group missed significantly more injections compared to the all patient choice group (33.3% vs 7.1%, respectively, p = 0.042).	*Adherence and therapeutic effects:* Over the 3-year period, height SDS and IGF-1 SDS were significantly higher in the all patient choice group (height SDS = 1.34 ± 0.44, p = 0.020; IGF-1 SDS = 2.49 ± 0.75, p = 0.038) compared to those in the non-patient choice group (height SDS = 0.92 ± 0.57; IGF-1 SDS = 1.89 ± 1.13).
Wickramasuriya et al.[86]	Compliance assess in 50 children who received GH by hospital prescription and home delivery and in whom uptake of ampoules could be determined:<br><br>For the 50 patients, the median compliance for all devices was 95% (range 84-105%), with 96% (range 93-100%) for needle-free devices and 87% (range 84-105%) for needled devices.<br><br>This compares to a median compliance of 88% for needle-free devices (only 1 device available) and 91% *(range 86-101%) (3 devices)) for needled devices for those [patients (n=115) who had not been offered free choice of GH device but were having hospital prescription with home delivery.	125 patients were offered free choice of device: 68 (54%) chose a needled device [pen devices; n = 65 or needle and syringe; n = *3*], and 57 (46%) needle-free.<br><br>Of the 50 patients, 22 (44%) chose a needle-free device, the remainder (28 (56%)) a needled device.

Abbreviations
[M] Male; [F] Female; [GHD] Growth Hormone Deficiency; Growth Hormone Insufficiency [GHI]; [SGA] Small for Gestational Age; [TS] Turner Syndrome; [rhGH], Recombinant Human Growth Hormone; [IGF-1] Insulin-like Growth Factor I; [SDS] Standard Deviation Score; [BMI] Body Mass Index; [CRF] Chronic Renal Failure; [HTSDS] Height Standard Deviation Scores; [MPHSDS-HTSDS] Mid-Parental Height Standard Deviation Scores; [PDC] Proportion of Days Covered. Needle Free Delivery System [*NFDS*]

Key
** Pubertal status unavailable for 5 patients.

References

1. Amereller, F.; Schilbach, K.; Schopohl, J.; Stormann, S. Adherence, Attitudes and Beliefs of Growth Hormone Deficient Patients - A Questionnaire-based Cohort Study. *Exp Clin Endocrinol Diabetes* **2021**, *129*, 112-117, doi:10.1055/a-0956-1919.
2. Assefi A; Chareca C; Roca F; Celis Ayala L; Rubstein A; Von Schulz Hausmann C. Positive impact on adherence through educational activities of the Argentina patient support program in children with low adherence to treatment with recombinant growth hormone (easypod connect electronic autoinjector device). Presented at ESPE 2019, 19–21 September, 2019; Vienna, Austria.
3. Assefi, A.R.; Roca, F.; Rubstein, A.; Chareca, C. Positive Impact of Targeted Educational Intervention in Children With Low Adherence to Growth Hormone Treatment Identified by Use of the Easypod Electronic Auto-injector Device. *Front Med Technol* **2021**, *3*, 609878, doi:10.3389/fmedt.2021.609878.
4. Aydin, B.K.; Aycan, Z.; Siklar, Z.; Berberoglu, M.; Ocal, G.; Cetinkaya, S.; Bas, V.N.; Kendirci, H.N.; Cetinkaya, E.; Darcan, S.; et al. Adherence to growth hormone therapy: results of a multicenter study. *Endocr Pract* **2014**, *20*, 46-51, doi:10.4158/EP13194.OR.
5. Ayuk L; Casey A; Prior J; Kirk J. Growth hormone device change-over; is it beneficial? Presented at the 41st Meeting of the British Society for Paediatric Endocrinology and Diabetes, Brighton UK. *Endocrine Abstracts* **2013**, *33*, P70.
6. Bagnasco, F.; Di Iorgi, N.; Roveda, A.; Gallizia, A.; Haupt, R.; Maghnie, M.; Adherence Investigators Group. Prevalence and Correlates of Adherence in Children and Adolescents Treated with Growth Hormone: A Multicenter Italian Study. *Endocr Pract* **2017**, *23*, 929-941, doi:10.4158/EP171786.OR.
7. Bercu, B.B.; Murray, F.T.; Frasier, S.D.; Rudlin, C.; O'Dea, L.S.; Brentzel, J.; Hanson, B.; Landy, H. Long-term therapy with recombinant human growth hormone (Saizen) in children with idiopathic and organic growth hormone deficiency. *Endocrine* **2001**, *15*, 43-49, doi:10.1385/endo:15:1:043.
8. Bozzola, M.; Pagani, S.; Iughetti, L.; Maffeis, C.; Bozzola, E.; Meazza, C. Adherence to growth hormone therapy: a practical approach. *Horm Res Paediatr* **2014**, *81*, 331-335, doi:10.1159/000357975.
9. Brearley, C.; Priestley, A.; Leighton-Scott, J.; Christen, M. Pharmacokinetics of recombinant human growth hormone administered by cool.click 2, a new needle-free

device, compared with subcutaneous administration using a conventional syringe and needle. *BMC Clin Pharmacol* **2007**, *7*, 10, doi:10.1186/1472-6904-7-10.

10. Choi H-J. Improving adherence to growth hormone (GH) therapy via easypod^{TM} may help maximize the treatment outcome. *Journal of Mucopolysaccharidosis and Rare Diseases* **2015**, *1*, 19-22.
11. Coutant, R.; Dupuis, C.; Pigeon, P.; Rebaud, P. Patients' perceptions on the usability of the SurePal self-injection device for Omnitrope((R)): a questionnaire-based observational study conducted in paediatric patients in France. *Ther Adv Endocrinol Metab* **2017**, *8*, 129-137, doi:10.1177/2042018817730545.
12. Cutfield, W.; Lindberg, A.; Albertsson Wikland, K.; Chatelain, P.; Ranke, M.B.; Wilton, P. Final height in idiopathic growth hormone deficiency: the KIGS experience. KIGS International Board. *Acta Paediatr Suppl* **1999**, *88*, 72-75, doi:10.1111/j.1651-2227.1999.tb14356.x.
13. Cutfield, W.S.; Derraik, J.G.; Gunn, A.J.; Reid, K.; Delany, T.; Robinson, E.; Hofman, P.L. Non-compliance with growth hormone treatment in children is common and impairs linear growth. *PLoS One* **2011**, *6*, e16223, doi:10.1371/journal.pone.0016223.
14. Dahlgren, J.; Veimo, D.; Johansson, L.; Bech, I. Patient acceptance of a novel electronic auto-injector device to administer recombinant human growth hormone: results from an open-label, user survey of everyday use. *Curr Med Res Opin* **2007**, *23*, 1649-1655, doi:10.1185/030079907x210589.
15. Dahlgren, J. Easypod: a new electronic injection device for growth hormone. *Expert Rev Med Devices* **2008**, *5*, 297-304, doi:10.1586/17434440.5.3.297.
16. De Pedro, S.; Murillo, M.; Salinas, I.; Granada, M.L.; Martinez, M.; Puig-Domingo, M.; Andreu, A.; Bel, J. Variability in adherence to rhGH treatment: Socioeconomic causes and effect on children's growth. *Growth Horm IGF Res* **2016**, *26*, 32-35, doi:10.1016/j.ghir.2015.12.002.
17. Dorr, H.G.; Zabransky, S.; Keller, E.; Otten, B.J.; Partsch, C.J.; Nyman, L.; Gillespie, B.K.; Lester, N.R.; Wilson, A.M.; Hyren, C.; et al. Are needle-free injections a useful alternative for growth hormone therapy in children? Safety and pharmacokinetics of growth hormone delivered by a new needle-free injection device compared to a fine gauge needle. *J Pediatr Endocrinol Metab* **2003**, *16*, 383-392, doi:10.1515/jpem.2003.16.3.383.

18. Dumas, H.; Panayiotopoulos, P.; Parker, D.; Pongpairochana, V. Understanding and meeting the needs of those using growth hormone injection devices. *BMC Endocr Disord* **2006**, *6*, 5, doi:10.1186/1472-6823-6-5.
19. Farfel, A.; Shalitin, S.; Morag, N.; Meyerovitch, J. Long-term adherence to growth hormone therapy in a large health maintenance organization cohort. *Growth Horm IGF Res* **2019**, *44*, 1-5, doi:10.1016/j.ghir.2018.10.004.
20. Fuchs, G.S.; Mikkelsen, S.; Knudsen, T.K.; Kappelgaard, A.M. Ease of use and acceptability of a new pen device for the administration of growth hormone therapy in pediatric patients: an open-label, uncontrolled usability test. *Clin Ther* **2009**, *31*, 2906-2914, doi:10.1016/j.clinthera.2009.12.014.
21. Graham, S.; Auyeung, V.; Weinman, J. Exploring Potentially Modifiable Factors That Influence Treatment Non-Adherence Amongst Pediatric Growth Hormone Deficiency: A Qualitative Study. *Patient Prefer Adherence* **2020**, *14*, 1889-1899, doi:10.2147/PPA.S268972.
22. Graham, S.; Neo, S.; Auyeung, V.; Weinman, J. What Potentially Modifiable Factors are Associated With Treatment Nonadherence in Pediatric Growth Hormone Deficiency? A Quantitative Study. *Endocr Pract* **2021**, *27*, 146-151, doi:10.4158/EP-2020-0543.
23. Haverkamp, F.; Johansson, L.; Dumas, H.; Langham, S.; Tauber, M.; Veimo, D.; Chiarelli, F. Observations of nonadherence to recombinant human growth hormone therapy in clinical practice. *Clin Ther* **2008**, *30*, 307-316, doi:10.1016/j.clinthera.2008.02.017.
24. Hey-Hadavi, J.; Pleil, A.; Deeb, L.C.; Fuqua, J.S.; Silverman, L.A.; Reiner, B.; Newfield, R.; Rajicic, N.; Wajnrajch, M.P.; Cara, J.F. Ease of use and preference for a new disposable self-injection pen compared with a reusable pen for administering recombinant human growth hormone: A multicenter, 2-month, single-arm, open-label clinical trial in patient-caregiver dyads. *Clin Ther* **2010**, *32*, 2036-2047, doi:10.1016/j.clinthera.2010.11.007.
25. Hindmarsh, P.C.; Brook, C.G. Compliance with growth hormone treatment - is it a problem? *Horm Res* **1999**, *51 Suppl 3*, 104-108, doi:10.1159/000053170.
26. Hokken-Koelega, A.; Keller, A.; Rakov, V.; Kipper, S.; Dahlgren, J. Patient Acceptance, Ease of Use, and Preference for Norditropin NordiFlex with NordiFlex PenMate: Results from an Open-Label, User Survey of Everyday Use. *ISRN Endocrinol* **2011**, *2011*, 803948, doi:10.5402/2011/803948.

27. Johannsson, G. Long-acting growth hormone for replacement therapy. *J Clin Endocrinol Metab* **2011**, *96*, 1668-1670, doi:10.1210/jc.2011-0689.
28. Kale TR; Momin M. Needle free injection technology - An overview. *Innov Pharm* **2014**, *5*.
29. Kapoor, R.; Burke, S.A.; Sparrow, S.; Hughes, I.; Dunger, D.; Ong, K.; Acerini, C. Monitoring of concordance in growth hormone therapy. *Archives of disease in childhood* **2008**, *93*, 147-148, doi:10.1136/adc.2006.114249.
30. Kappelgaard, A.M.; Mikkelsen, S.; Knudsen, T.K.; Fuchs, G.S. Patient preference for a new growth hormone injection device: results of an open-label study in Japanese pediatric patients. *J Pediatr Endocrinol Metab* **2011**, *24*, 489-496, doi:10.1515/jpem.2011.252.
31. Kappelgaard, A.M.; Mikkelsen, S.; Bagger, C.; Fuchs, G.S. Children and adolescent acceptability of a new device system to administer human growth hormone--a pilot study. *J Pediatr Endocrinol Metab* **2012**, *25*, 285-294, doi:10.1515/jpem-2011-0395.
32. Kappelgaard, A.M.; Hansen, N.A. Comparison of injection dose force, accuracy and precision among three growth hormone injection devices. *Expert Rev Med Devices* **2013**, *10*, 321-327, doi:10.1586/erd.13.12.
33. Kappelgaard, A.M.; Laursen, T. The benefits of growth hormone therapy in patients with Turner syndrome, Noonan syndrome and children born small for gestational age. *Growth Horm IGF Res* **2011**, *21*, 305-313, doi:10.1016/j.ghir.2011.09.004.
34. Kappelgaard, A.M.; Metzinger, C.P.; Schnabel, D. A web-based survey assessing the impact of storage flexibility on the daily life of patients and caregivers administering growth hormone. *Expert Rev Med Devices* **2015**, *12*, 517-527, doi:10.1586/17434440.2015.1069180.
35. Kaptein, A.A. Transjecting growth hormone: continuous nightmare or controlled nuisance? Evaluation of a new needle-free device. *Patient Prefer Adherence* **2013**, *7*, 703-708, doi:10.2147/PPA.S46990.
36. Khadilkar, V.; Radjuk, K.A.; Bolshova, E.; Khadgawat, R.; El Kholy, M.; Desai, M.; Peterkova, V.; Mericq, V.; Kratzsch, J.; Siepl, E.C.; et al. 24-month use of once-weekly GH, LB03002, in prepubertal children with GH deficiency. *J Clin Endocrinol Metab* **2014**, *99*, 126-132, doi:10.1210/jc.2013-2502.
37. Koledova, E.; Stoyanov, G.; Ovbude, L.; Davies, P.S.W. Adherence and long-term growth outcomes: results from the easypod() connect observational study (ECOS) in

paediatric patients with growth disorders. *Endocr Connect* **2018**, *7*, 914-923, doi:10.1530/EC-18-0172.

38. Lass, N.; Reinehr, T. Low Treatment Adherence in Pubertal Children Treated with Thyroxin or Growth Hormone. *Horm Res Paediatr* **2015**, *84*, 240-247, doi:10.1159/000437305.
39. Lopez Siguero, J.P.; Martinez Aedo, M.J.; Lopez Moreno, M.D.; Martinez Valverde, A. Treatment with growth hormone. What do children know and how do they accept it? *Horm Res* **1995**, *44 Suppl 3*, 18-25, doi:10.1159/000184669.
40. Luo, X.; Hou, L.; Liang, L.; Dong, G.; Shen, S.; Zhao, Z.; Gong, C.X.; Li, Y.; Du, M.L.; Su, Z.; et al. Long-acting PEGylated recombinant human growth hormone (Jintrolong) for children with growth hormone deficiency: phase II and phase III multicenter, randomized studies. *Eur J Endocrinol* **2017**, *177*, 195-205, doi:10.1530/EJE-16-0905.
41. Lustig, R.H. Optimizing growth hormone efficacy: an evidence-based analysis. *Horm Res* **2004**, *62 Suppl 3*, 93-97, doi:10.1159/000080506.
42. Main, K.M.; Jorgensen, J.T.; Hertel, N.T.; Jensen, S.; Jakobsen, L. Automatic needle insertion diminishes pain during growth hormone injection. *Acta Paediatr* **1995**, *84*, 331-334, doi:10.1111/j.1651-2227.1995.tb13638.x.
43. Meinhardt, U.; Eiholzer, U.; Seitz, L.; Bogelund, M.; Kappelgaard, A.M. Parent preference in Switzerland for easy-to-use attributes of growth hormone injection devices quantified by willingness to pay. *Expert Rev Med Devices* **2014**, *11*, 31-38, doi:10.1586/17434440.2014.856754.
44. Miller BS; Rotenstein D; Deeb LC; Germak J; Wisniewski T. Persistence with growth hormone therapy in pediatric patients. *Am J Pharm Benefits* **2014**, *6*, e9-e17.
45. Mitragotri, S. Current status and future prospects of needle-free liquid jet injectors. *Nat Rev Drug Discov* **2006**, *5*, 543-548, doi:10.1038/nrd2076.
46. Mohseni, S.; Heydari, Z.; Qorbani, M.; Radfar, M. Adherence to growth hormone therapy in children and its potential barriers. *J Pediatr Endocrinol Metab* **2018**, *31*, 13-20, doi:10.1515/jpem-2017-0157.
47. Moore, W.V.; Nguyen, H.J.; Kletter, G.B.; Miller, B.S.; Rogers, D.; Ng, D.; Moore, J.A.; Humphriss, E.; Cleland, J.L.; Bright, G.M. A Randomized Safety and Efficacy Study of Somavaratan (VRS-317), a Long-Acting rhGH, in Pediatric Growth Hormone Deficiency. *J Clin Endocrinol Metab* **2016**, *101*, 1091-1097, doi:10.1210/jc.2015-3279.

48. Muller, J.; Skakkebaek, N.E.; Jacobsen, B.B.; Keller, E.; Heinrich, U.; Hartmann, K.; Hokken-Koelega, A.C.; Delemarre van de Waal, H.A. Norditropin SimpleXx: a liquid human growth hormone formulation, a pen system and an auto-insertion device. *Horm Res* **1999**, *51 Suppl 3*, 109-112, doi:10.1159/000053171.
49. Nicolino M; Coutant R; Tauber M; Ribault V; Lopez Y; Besserve A; van Hille B. PMD23 - Easypod™ Connect Observational Study (ECOS) – French case histories and growth outcomes. *Value Health* **2018**, *21*, S246.
50. Norgren, S. Adherence remains a challenge for patients receiving growth hormone therapy. *Pediatr Endocrinol Rev* **2009**, *6 Suppl 4*, 545-548.
51. Osterberg, L.; Blaschke, T. Adherence to medication. *N Engl J Med* **2005**, *353*, 487-497, doi:10.1056/NEJMra050100.
52. Oyarzabal, M.; Aliaga, M.; Chueca, M.; Echarte, G.; Ulied, A. Multicentre survey on compliance with growth hormone therapy: what can be improved? *Acta Paediatr* **1998**, *87*, 387-391, doi:10.1080/08035259850156959.
53. Partsch, C.J.; Schnabel, D.; Ehtisham, S.; Johnstone, H.C.; Zabransky, M.; Kiess, W. Acceptability of the reusable SurePal self-injection device for Omnitrope((R)) among pediatric patients: results from a questionnaire-based, cross-sectional, multicenter observational study. *Med Devices (Auckl)* **2015**, *8*, 389-393, doi:10.2147/MDER.S93209.
54. Pfutzner, A.; Hartmann, K.; Winter, F.; Fuchs, G.S.; Kappelgaard, A.M.; Rohrer, T.R. Intuitiveness, ease of use, and preference of a prefilled growth hormone injection pen: a noninterventional, randomized, open-label, crossover, comparative usability study of three delivery devices in growth hormone-treated pediatric patients. *Clin Ther* **2010**, *32*, 1918-1934, doi:10.1016/j.clinthera.2010.10.010.
55. Pleil, A.M.; Darendeliler, F.; Dorr, H.G.; Hutchinson, K.; Wollmann, H.A. Results from an international multicenter trial evaluating the ease-of-use of and preference for a newly developed disposable injection pen for the treatment of growth hormone deficiency in treatment-naive children and adults. *Med Devices (Auckl)* **2014**, *7*, 61-71, doi:10.2147/MDER.S59821.
56. Rapaport, R.; Saenger, P.; Schmidt, H.; Hasegawa, Y.; Colle, M.; Loche, S.; Marcantonio, S.; Bonfig, W.; Zabransky, M.; Lifshitz, F. Validation and ease of use of a new pen device for self-administration of recombinant human growth hormone: results from a two-center usability study. *Med Devices (Auckl)* **2013**, *6*, 141-146, doi:10.2147/MDER.S50088.

57. Rosenfeld, R.G.; Bakker, B. Compliance and persistence in pediatric and adult patients receiving growth hormone therapy. *Endocr Pract* **2008**, *14*, 143-154, doi:10.4158/EP.14.2.143.
58. Sauer, M.; Abbotts, C. A new pen device for injection of recombinant human growth hormone: a convenience, functionality and usability evaluation study. *Patient Prefer Adherence* **2018**, *12*, 27-34, doi:10.2147/PPA.S149412.
59. Shine, B.; Musial, W.; Owens, L.; Deeb, L.; Luetjen, T.; Howard, C. Patient and parent preference for growth hormone products. *Am J Health Syst Pharm* **2003**, *60*, 89-90, doi:10.1093/ajhp/60.1.89.
60. Silverstein, J.H.; Murray, F.T.; Malasanos, T.; Myers, S.; Johnson, S.B.; Frye, K.; Grossman, M. Clinical testing results and high patient satisfaction with a new needle-free device for growth hormone in young children. *Endocrine* **2001**, *15*, 15-17, doi:10.1385/ENDO:15:1:015.
61. Sjoblom, K.; Albertsson-Wikland, K.; Bengtsson, B.A.; Johannsson, G.; Thoren, M.; Degerblad, M.; Savage, M.O. Patient evaluation of a new injection pen for growth hormone treatment in children and adults. *Acta Paediatr Suppl* **1995**, *411*, 63-65, doi:10.1111/j.1651-2227.1995.tb13867.x.
62. Smith, S.L.; Hindmarsh, P.C.; Brook, C.G. Compliance with growth hormone treatment--are they getting it? *Arch Dis Child* **1993**, *68*, 91-93, doi:10.1136/adc.68.1.91.
63. Stanhope, R.; Albanese, A.; Moyle, L.; Hamill, G. Optimum method for administration of biosynthetic human growth hormone: a randomised crossover trial of an Auto Injector and a pen injection system. *Arch Dis Child* **1992**, *67*, 994-997, doi:10.1136/adc.67.8.994.
64. Stanhope, R.; Moyle, L.; MacSwiney, M. Patient knowledge and compliance with growth hormone treatment. *Arch Dis Child* **1993**, *68*, 525, doi:10.1136/adc.68.4.525.
65. Staples, B.; Bravender, T. Drug compliance in adolescents: assessing and managing modifiable risk factors. *Paediatr Drugs* **2002**, *4*, 503-513, doi:10.2165/00128072-200204080-00003.
66. Tauber, M.; Payen, C.; Cartault, A.; Jouret, B.; Edouard, T.; Roger, D. User trial of Easypod, an electronic autoinjector for growth hormone. *Ann Endocrinol (Paris)* **2008**, *69*, 511-516, doi:10.1016/j.ando.2008.04.003.

67. Tebbi, C.K. Treatment compliance in childhood and adolescence. *Cancer* **1993**, *71*, 3441-3449, doi:10.1002/1097-0142(19930515)71:10+<3441::aid-cncr2820711751>3.0.co;2-p.
68. Tollerfield, S.; Criseno, S.; Fallon, M.; Jennings, C.; Jones, J.; Marland, A.; Martin, L.; Ward, S.; Whitehead, A. Facilitating the adherence journey of children, adolescents, and adults on long-term growth hormone therapy. *Br J Nurs* **2020**, *29*, 1118-1123, doi:10.12968/bjon.2020.29.19.1118.
69. Verrips, G.H.; Hirasing, R.A.; Fekkes, M.; Vogels, T.; Verloove-Vanhorick, S.P.; Delemarre-Van de Waal, H.A. Psychological responses to the needle-free Medi-Jector or the multidose Disetronic injection pen in human growth hormone therapy. *Acta Paediatr* **1998**, *87*, 154-158, doi:10.1080/08035259850157589.
70. Weill J; Niez P. Adherence to the treatment with Zomajet™, a needle-free device transjecting growth hormone: results of French observational survey (P2-d1-961). Poster presented at the European Society for Paediatric Endocrinology Annual Conference, September 19-22, 2013, Milan, Italy.
71. Zamora Ferrer E; Canales Ugarte S; Nieto-Sandoval Martín de La Sierra P; Heredia Benito M; Gómez Lluch T; Valenzuela Gámez JC. DI-059 Pharmacotherapeutic monitoring in growth-hormone treatment adherence. *Eur J Hosp Pharm* **2015**, *22*, A97-A98.
72. Arrabal Vela, M.A.; Garcia Gijon, C.P.; Pascual Martin, M.; Benet Gimenez, I.; Areas Del Aguila, V.; Munoz-Rodriguez, J.R.; Palomo Atance, E. Adherence to somatotropin treatment administered with an electronic device. *Endocrinol Diabetes Nutr (Engl Ed)* **2018**, *65*, 314-318, doi:10.1016/j.endinu.2018.02.003.
73. Blanco-Lopez, A.; Antillon-Ferreira, C.; Saavedra-Castillo, E.; Barrientos-Perez, M.; Rivero-Escalante, H.; Flores-Caloca, O.; Calzada-Leon, R.; Rosas-Guerra, C.C.; Koledova, E.; Chiquete, E.; et al. Adherence to treatment in children with growth hormone deficiency, small for gestational age and Turner syndrome in Mexico: results of the Easypod connect observational study (ECOS). *J Endocrinol Invest* **2020**, *43*, 1447-1452, doi:10.1007/s40618-020-01218-4.
74. Bozzola, M.; Colle, M.; Halldin-Stenlid, M.; Larroque, S.; Zignani, M.; easypod survey study, g. Treatment adherence with the easypod growth hormone electronic auto-injector and patient acceptance: survey results from 824 children and their parents. *BMC Endocr Disord* **2011**, *11*, 4, doi:10.1186/1472-6823-11-4.

75. Centonze, C.; Guzzetti, C.; Orlando, G.; Loche, S.; Italian, E.I. Adherence to growth hormone (GH) therapy in naive to treatment GH-deficient children: data of the Italian Cohort from the Easypod Connect Observational Study (ECOS). *J Endocrinol Invest* **2019**, *42*, 1241-1244, doi:10.1007/s40618-019-01046-1.
76. Hartmann, K.; Ittner, J.; Muller-Rossberg, E.; Schonau, E.; Stephan, R.; Ullrich, K.P.; Hoppe, B.; Ramseger, R.; Bramswig, J. Growth hormone treatment adherence in prepubertal and pubertal children with different growth disorders. *Horm Res Paediatr* **2013**, *80*, 1-5, doi:10.1159/000351800.
77. Loche, S.; Salerno, M.; Garofalo, P.; Cardinale, G.M.; Licenziati, M.R.; Citro, G.; Caruso Nicoletti, M.; Cappa, M.; Longobardi, S.; Maghnie, M.; et al. Adherence in children with growth hormone deficiency treated with r-hGH and the easypod device. *J Endocrinol Invest* **2016**, *39*, 1419-1424, doi:10.1007/s40618-016-0510-0.
78. Maggio, M.C.; Vergara, B.; Porcelli, P.; Corsello, G. Improvement of treatment adherence with growth hormone by easypod device: experience of an Italian centre. *Ital J Pediatr* **2018**, *44*, 113, doi:10.1186/s13052-018-0548-z.
79. Rodríguez Arnao MD; Rodríguez Sánchez A; Díez López I; Ramírez Fernández J; Bermúdez de la Vega JA; Yeste Fernández D; Chueca Guindulain M; Corripio Collado R; Pérez Sánchez J; Fernández González A; et al. Adherence and long-term outcomes of growth hormone therapy with easypod™ in pediatric subjects: Spanish ECOS study. *Endocr Connect* **2019**, *8*, 1240-1249.
80. van Dommelen P; Koledova E; Wit JM. Effect of adherence to growth hormone treatment on 0–2 year catch-up growth in children with growth hormone deficiency. *PLoS One* **2018**, *13*, e0206009.
81. Desrosiers, P.; O'Brien, F.; Blethen, S. Patient outcomes in the GHMonitor: the effect of delivery device on compliance and growth. *Pediatr Endocrinol Rev* **2005**, *2 Suppl 3*, 327-331.
82. Michaelidou, M.; Whitten, S.; Bajaj, P.; Knight, A.; Spoudeas, H.A. Improved adherence and growth outcomes with jet-delivered growth hormone. *J Pediatr Endocrinol Metab* **2019**, *32*, 207-213, doi:10.1515/jpem-2018-0067.
83. Spoudeas, H.A.; Bajaj, P.; Sommerford, N. Maintaining persistence and adherence with subcutaneous growth-hormone therapy in children: comparing jet-delivery and needle-based devices. *Patient Prefer Adherence* **2014**, *8*, 1255-1263, doi:10.2147/PPA.S70019.

84. Tauber, M.; Jaquet, D.; Jesuran-Perelroizen, M.; Petrus, M.; Bertrand, A.M.; Coutant, R.; NordiFlex French Study, G. User assessment of Norditropin NordiFlex((R)), a new prefilled growth hormone pen: a Phase IV multicenter prospective study. *Patient Prefer Adherence* **2013**, *7*, 455-462, doi:10.2147/PPA.S43460.
85. Gau, M.; Takasawa, K. Initial patient choice of a growth hormone device improves child and adolescent adherence to and therapeutic effects of growth hormone replacement therapy. *J Pediatr Endocrinol Metab* **2017**, *30*, 989-993, doi:10.1515/jpem-2017-0146.
86. Wickramasuriya, B.P.; Casey, A.; Akhtar, S.; Zia, R.; Ehtisham, S.; Barrett, T.G.; Shaw, N.J.; Kirk, J.M. Factors determining patient choice of device for GH therapy. *Horm Res* **2006**, *65*, 18-22, doi:10.1159/000090375.
